# Efficacy, immunogenicity and safety of COVID-19 vaccines in older adults: a systematic review and meta-analysis

**DOI:** 10.3389/fimmu.2022.965971

**Published:** 2022-09-13

**Authors:** Zejun Li, Shouhuan Liu, Fengming Li, Yifeng Li, Yilin Li, Pu Peng, Sai Li, Li He, Tieqiao Liu

**Affiliations:** ^1^ National Clinical Research Center for Mental Disorders, Department of Psychiatry, The Second Xiangya Hospital of Central South University, Changsha, China; ^2^ Department of Psychiatry, First Affiliated Hospital of Kunming Medical University, Kunming, China; ^3^ Ministry of Education Key Laboratory of Child Development and Disorders, Children’s Hospital of Chongqing Medical University, Chongqing, China; ^4^ College of Pediatrics, Chongqing Medical University, Chongqing, China

**Keywords:** COVID-19 vaccines, efficacy, immunogenicity, safety, older adults, randomized controlled trials (RCTs), meta-analysis

## Abstract

**Background:**

Older adults are more susceptible to severe health outcomes for coronavirus disease 2019 (COVID-19). Universal vaccination has become a trend, but there are still doubts and research gaps regarding the COVID-19 vaccination in the elderly. This study aimed to investigate the efficacy, immunogenicity, and safety of COVID-19 vaccines in older people aged ≥ 55 years and their influencing factors.

**Methods:**

Randomized controlled trials from inception to April 9, 2022, were systematically searched in PubMed, EMBASE, the Cochrane Library, and Web of Science. We estimated summary relative risk (RR), rates, or standardized mean difference (SMD) with 95% confidence interval (CI) using random-effects meta-analysis. This study was registered with PROSPERO (CRD42022314456).

**Results:**

Of the 32 eligible studies, 9, 21, and 25 were analyzed for efficacy, immunogenicity, and safety, respectively. In older adults, vaccination was efficacious against COVID-19 (79.49%, 95% CI: 60.55−89.34), with excellent seroconversion rate (92.64%, 95% CI: 86.77−96.91) and geometric mean titer (GMT) (SMD 3.56, 95% CI: 2.80−4.31) of neutralizing antibodies, and provided a significant protection rate against severe disease (87.01%, 50.80−96.57). Subgroup and meta-regression analyses consistently found vaccine types and the number of doses to be primary influencing factors for efficacy and immunogenicity. Specifically, mRNA vaccines showed the best efficacy (90.72%, 95% CI: 86.82−93.46), consistent with its highest seroconversion rate (98.52%, 95% CI: 93.45−99.98) and GMT (SMD 6.20, 95% CI: 2.02−10.39). Compared to the control groups, vaccination significantly increased the incidence of total adverse events (AEs) (RR 1.59, 95% CI: 1.38−1.83), including most local and systemic AEs, such as pain, fever, chill, etc. For inactivated and DNA vaccines, the incidence of any AEs was similar between vaccination and control groups (*p* > 0.1), while mRNA vaccines had the highest risk of most AEs (RR range from 1.74 to 7.22).

**Conclusion:**

COVID-19 vaccines showed acceptable efficacy, immunogenicity and safety in older people, especially providing a high protection rate against severe disease. The mRNA vaccine was the most efficacious, but it is worth surveillance for some AEs it caused. Increased booster coverage in older adults is warranted, and additional studies are urgently required for longer follow-up periods and variant strains.

## Introduction

Coronavirus disease 2019 (COVID-19), caused by severe acute respiratory syndrome coronavirus 2 (SARS-CoV-2), has emerged as a global pandemic and posed a considerable threat to global public health and the global economy. As of June 8, 2022, over 530 million confirmed cases and over 6.3 million deaths had been reported worldwide (1). Older adults, many of whom often have chronic health conditions (2, 3), have a higher prevalence of COVID-19 and a more elevated risk of severe illness and death compared to younger persons (4–9). Meanwhile, a new study conducted by Imperial College London found that the SARS-CoV-2 infection rate was still rising among the older adults (i.e., aged ≥ 55 years) in the UK, while this was not seen in the younger population (10). Therefore, effective measures to protect these vulnerable older adults are urgently needed.

An effective vaccine against SARS-CoV-2 is one of the most important measures to control the global COVID-19 pandemic, significantly reducing the risk of asymptomatic infection and progression to severe clinical outcomes in the elderly (4, 11–14). As of June 7, 2022, there were 163 candidate vaccines in clinical development and 198 in pre-clinical development, including the following main categories: viral vector vaccines, inactivated virus vaccines, subunit vaccines, DNA- and mRNA-based vaccines, as well as virus-like particle vaccines, etc. (15) It is encouraging that the COVID-19 vaccines have been developed at such an astonishing pace in just two years. However, with this comes a variety of concerns about the vaccines; for instance, to what extent can they reduce the risk of infection in older adults? How often are the adverse events happening to the older adults after vaccination? How serious are they? These topics deserve persistent and extensive surveillance and research (16), as they are the main reasons for COVID-19 vaccine hesitancy (17, 18). And these hesitant factors contribute to a lower rate of willingness to receive the COVID-19 vaccines in older adults than in adults aged 18-59 years (18). Accordingly, providing scientific evidence to effectively dispel the various concerns of the elderly and their offspring is an urgently warranted issue.

Most randomized clinical trials (RCTs) have shown varying degrees of local and systemic adverse events in participants after vaccination and demonstrated efficacy in reducing the risk of SARS-CoV-2 infection (19–25). And there are apparent distinctions in efficacy and safety among all kinds of vaccines (26–28). COVID-19 vaccines may work differently in the elderly population than in other adults due to age-related immune impairment (29). Some studies have indicated that vaccination safety in older adults was probably better than that in younger adults, but the immune effect was probably worse (11, 30–32). Besides, although clinical studies of COVID-19 vaccines have progressed to phase 3 and even phase 4 (15, 23, 33), it has been reported that the RCTs for COVID-19 probably excluded older persons (34, 35). Therefore, more high-quality studies and robust information are required to determine each vaccine’s efficacy, immunogenicity and safety in older people and confirm which vaccine type was the most optimal (36). Previous meta-analyses have included a limited number of studies, sample sizes, and vaccine types (37–44). And these studies were all based on adult populations ≥ 18 years old and specific evidences for the efficacy, immunogenicity and safety of COVID-19 vaccines in older populations are still lacking. Consequently, this study aims to comprehensively integrate and evaluate the efficacy, immunogenicity and safety of the COVID-19 vaccines in the elderly, based on the existing RCTs to provide reliable evidence and reference for further in-depth studies on COVID-19 vaccines and for the elderly who remain apprehensive about the vaccination.

## Methods

This systematic review and meta-analysis was reported in accordance with the Preferred Reporting Items for Systematic Reviews and Meta-Analyses (PRISMA) statement (45), and the study protocol was registered on PROSPERO (CRD42022314456).

### Search strategy

PubMed, EMBASE, the Cochrane Central Register of Controlled Trials, and Web of Science were systematically searched to identify literature published from inception to April 9, 2022. This study used a combination of Medical Subject Headings terms and free terms based on the following seven terms and their synonyms: older adults, COVID-19, vaccine, efficacy, immunogenicity, safety, and RCTs. Detailed search terms for each database were listed in the **Supplementary Materials**. The reports were limited to the English language, with no publication date restrictions.

### Study selection

All included original records should meet the following criteria: (1) randomized controlled trials (RCTs) evaluating the efficacy, immunogenicity, or safety of the COVID-19 vaccines in older adults; (2) studies containing cohorts with participants aged ≥ 55 years who were in good health or stable chronic health conditions and without a history of SARS-CoV-2 infection; (3) studies comparing various COVID-19 vaccines with control conditions that received a placebo, a non-vaccine alternative, or a vaccine other than SARS-CoV-2; (4) specific data on the outcomes of both vaccination and control groups were available; (5) peer-reviewed studies involving blinding. Exclusion criteria were as follows: (1) quasi-randomized trials; (2) non-original investigations: reviews, meta-analyses, letters, commentaries, editorials, errata, and other articles; (3) animal or *in vitro* model experiments and cell-line studies; (4) conference abstracts or studies where the authors had been contacted, but still unable to extract valid data or data on the elderly could not be extracted separately; (5) duplicate studies. When more than one report was from the same trial, the report containing the comprehensive data set was included to avoid data duplication.

**Figure 1:**
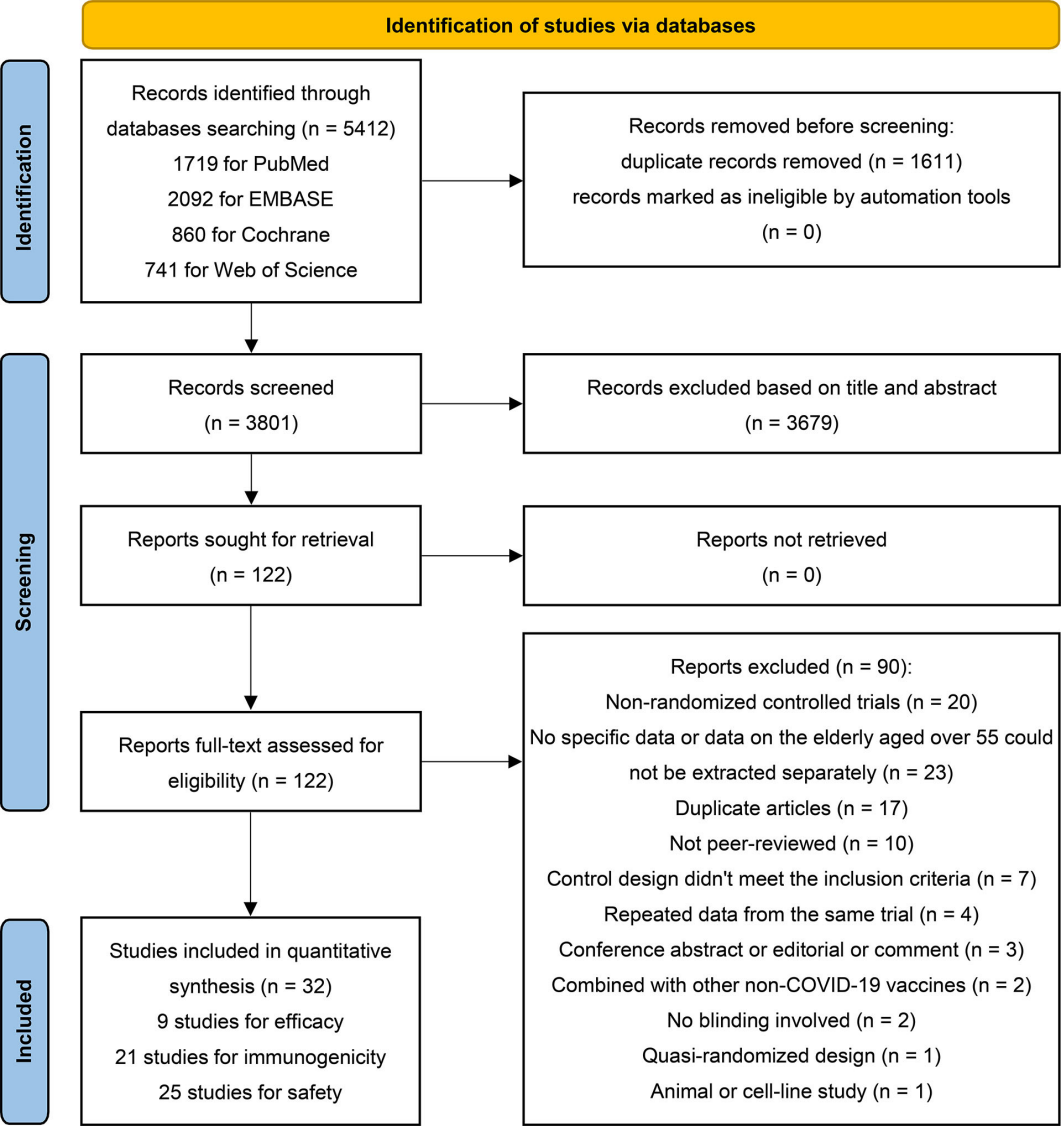
PRISMA 2020 flow diagram for identification of studies *via* databases.

Two investigators (Yf L and Yl L) independently screened the titles and abstracts, and potentially eligible studies were searched for full-text assessment. Any disagreements were determined by consensus and arbitrated by the third reviewers (TL and LH). Inter-rater reliability was assessed with kappa (score = 0.89). **Figure 1** illustrates the process for selecting eligible studies and the reasons for exclusion.

### Data extraction and quality assessment

Two investigators (SL and FL) independently extracted data and assessed the methodological quality of the eligible studies. Any disagreements were resolved by the third researchers (TL and LH). A standardized data abstraction form constructed with Microsoft Excel was used to record the extracted information, including (1) basic information: title, first author, date of publication, and the country conducting the studies; (2) study design: blinding, study phase and the number of study centers; (3) demographic information: age range, country of participants, race or ethnicity and sample size of each group; (4) general methodological details: original inclusion or exclusion criteria of participants and length of follow up; (5) details of intervention: types of vaccine and control intervention, vaccines, dosage, and the initiation and interval time of vaccination; (6) outcome information: outcomes of efficacy, immunogenicity and safety, and the time and method of outcomes measurement. We combined groups in studies with more than one intervention group when necessary (46). Study authors were contacted directly when detailed information on the outcomes was incomplete. Data extraction software (GetData Graph Digitizer 2.26) was used to extract data from the original literature.

We assessed the bias risk of eligible studies using the Cochrane Risk of Bias tool for RCTs. The evaluation criteria included the following seven domains: random sequence generation, allocation concealment, blinding of participants and personnel, blinding of outcome assessment, incomplete outcome data, selective reporting, and other bias. The risk of bias graph was created by Revman 5.4 software. The certainty of evidence for the primary outcomes was evaluated using the Grades of Recommendation, Assessment, Development, and Evaluation (GRADE) system across the following five domains: study limitations, imprecision, heterogeneity and inconsistency, indirectness, and publication bias (47).

### Outcomes

Our primary outcomes were (1) the vaccine efficacy (VE), defined as the percentage reduction of SARS-CoV-2 infections in the vaccinated group compared to the control group after the last vaccination dose within a certain observation period, specifically VE = (infection rate in the control group − infection rate in the vaccine group)/(infection rate in the control group) = 1 − RR, RR refers to the risk ratio; (2) the immunogenicity of the vaccines, defined as the neutralizing antibody geometric mean titer (GMT) and seroconversion rate at 14 to 28 days after the last vaccine administration; (3) the incidence of solicited adverse events (AEs) after every dose of vaccination, including local AEs, systemic AEs, any injection of local AEs (such as pain, redness, swelling), and any systemic AEs (such as fever, fatigue, headache, chills, vomiting or nausea, diarrhea, malaise, myalgia, arthralgia), and the risk of grade 3 or higher AEs. The second primary outcomes were (1) the vaccine efficacy for symptomatic and severe COVID-19; (2) the seroconversion rates of spike-specific and RBD-specific antibodies; (3) the comparison of the incidence of AEs between the first and second doses. COVID-19 was confirmed by positive reverse transcriptase PCR (RT-PCR) or similar laboratory tests. Seroconversion rate definitions were based on each original article.

### Data synthesis and analysis

We performed this meta-analysis in Stata17.0 software, and the subgroup analysis forest charts were made using Microsoft Excel. The standardized mean difference (SMD) of the log-transformed GMT and the corresponding 95% confidence interval (CI) were used to represent the immunogenicity differences between the vaccination and control groups. The SMD was calculated using the DerSimonian-Laird model based on the mean and standard deviation of the two groups. Missing standard deviation of each group was calculated based the confidence interval and sample size (46). The seroconversion rates were expressed as pooled rate estimates and 95% CI. When the rates in most of the included studies were too low (< 30%) or too high (> 70%), pooled rate estimates were calculated based on the transformed values of the double arcsine method, then back-transformed to the original rates (48). Other dichotomous variables were represented as relative risk (RR) and 95% CI. Studies without events in both the vaccination and control groups were excluded from the summary of RR (46). When only one of the two groups did not have an event, a fixed value (0.5) was added to each cell of the 2 × 2 table of the trial for correction (46). Galbraith radial plot and I^2^ statistic were used to indicate heterogeneity among the included studies. A random-effects meta-analysis was conducted when considerable statistical heterogeneity (I^2^ > 50% or *p* < 0.1).

We performed subgroup analyses and meta-regression to explore potential sources of heterogeneity using the following variables: country, continent, study phase, number of centers, blinding, literature quality (risk of bias), vaccine types, number of doses, outcome measurement time, follow-up time, and sample size. We used visual examinations of funnel plots and Egger’s test to assess potential publication bias. The trim-and-fill analysis was used to assess the effect of publication bias on the pooled effect size estimates. Influence analysis (a type of sensitivity analysis) was conducted to identify the impact of individual studies on the combined estimates. *P* < 0.05 means statistically significant in this study.

## Results

### Search results and study characteristics

A total of 5412 records were retrieved initially from the databases. After screening titles and abstracts, we assessed 122 potentially eligible reports in full text; ultimately, 32 studies were included (22, 49–79). The two primary reasons for study exclusion were the unavailability of outcomes data on the elderly population and the study design as non-RCTs. Of the 32 eligible studies, 9, 21, and 25 were used to quantify the efficacy, immunogenicity and safety of COVID-19 vaccines, respectively (**Figure 1**). Included studies reported data from five vaccine types: non-replicating adenovirus vector vaccines (n = 9), subunit vaccines (n = 8), inactivated virus vaccines (n = 7), mRNA vaccines (n = 7), and DNA vaccines (n = 1) (**Table 1**). One trial simultaneously investigated the safety of two mRNA vaccines (BNT162b1 and BNT162b2) (74). There were 4 studies on one-dose vaccination (all for adenovirus vector vaccines) (57, 69, 70, 78), 23 on two-dose vaccination, and 5 on three-dose vaccination (4 for inactivated virus vaccines, 1 for subunit vaccine) (23, 56, 61, 63, 66). Among the 28 studies that received two or more vaccine doses, most vaccination intervals were 14 to 28 days, and only two studies had an interval of more than one month (22, 79). Remarkably, except for one study with control of meningococcal vaccine (MenACWY) (67), all other studies had placebos as controls. Included studies involved about 20 countries or regions, where China conducted the most studies (n = 12), followed by the United States (n = 7). All eligible studies were phase I to III clinical trials, and no data from phase IV clinical trials were reported. Approximately one-third of the eligible studies were observer-blinded (n = 10) (51, 52, 55, 58, 59, 65, 67, 71, 73, 74), and two-thirds were double-blind (n = 22) (**Tables 2**, **3**, and **Table S1**). Additionally, the assessment results of the bias risk for individual studies are illustrated in **Figure S6**. Fifteen trials were rated high on risk of bias, 16 as unclear, and one as low.

**Table 1:** The number of studies used to quantify the efficacy, safety, and immunogenicity of COVID-19 vaccines.

Vaccine type	Total no. of studies	No. of doses	No. of studies for efficacy	No. of studies for immunogenicity	No. of studies for safety
Adenovirus vector vaccines (non-replicating)					
Ad26.COV2.S	2	1	1	1	1
Ad5-nCoV	3	1, 2	1	2	1
ChAdOx1-S-(AZD1222)	3	2	1	1	3
Gam-COVID-Vac	1	2	1	1	1
Subunit vaccines					
MVC-COV1901	1	2	0	1	1
NVX-CoV2373	2	2	1	1	1
Recombinant COVID-19 vaccine (Sf9 cells)	1	3	0	1	1
Recombinant SARS-CoV-2 Fusion Protein Vaccine (V-01)	2	2	0	2	2
SCB-2019	2	2	1	1	1
Inactivated virus vaccines					
BBV152 vaccine	1	2	1	1	0
BBIBP-CorV	1	2	0	1	1
CoronaVac	3	2, 3	0	3	3
KCONVAC	1	3	0	1	1
WIBP COVID-19 vaccine	1	3	0	1	1
mRNA vaccines					
BNT162b1	2	2	0	1	2
BNT162b2	3	2	1	0	2
mRNA-1273	3	2	1	2	3
DNA vaccines					
ZyCoV-D	1	3	0	0	1

There was a study that investigated two vaccines concurrently (BNT162b1 and BNT162b2).

**Table 2:** Characteristics of included studies on the efficacy of COVID-19 vaccines.

Study	Vaccine	Administration (no. of doses, intervals, dosage)	Age range	No. of participants (vaccination/control)	Country	Study types (phase, no. of centers, blinding)	Vaccine efficacy(95% CI)
Bravo 2022 (50)	SCB-2019	2, 21 d, 30 µg	≥ 65	121/127	Belgium, Brazil, Colombia, Philippines, South Africa	II/III, 31, double-blind	58.4% (−73.4, 92.9)†
Ella 2021* (53)	BBV152 vaccine	2, 28 d, 6 µg	≥ 60	893/965	Indian	III, 25, double-blind	67.8% (8.0, 90.0)
Falsey 2021 (54)	ChAdOx1-S- (AZD1222)	2, 28 d, 5 × 10^10^ VP	≥ 65	3696/1812	the United States, Chile, Peru	III, 88, double-blind	83.5% (54.2, 94.1)
Halperin 2022 (57)	Ad5-nCoV	1, −, 5 × 10^10^ VP	≥ 60	1323/1347	Pakistan, Mexico, Russia, Chile, Argentina	III, 66, double-blind	53.3% (0.9, 78.0)
Heath 2021 (59)	NVX-CoV 2373	2, 21 d, 5 µg	≥ 65	1953/1957	the UK	III, 33, observer-blinded	88.9% (12.8, 98.6)
Logunov 2021* (64)	Gam-COVID-Vac	2, 21 d, 1 × 10^11^ VP	> 60	1611/533	Russia	III, 25, double-blind	91.8% (67.1, 98.3)
Sadoff 2022 (70)	Ad26.COV2.S	1, −, 5 × 10^10^ VP	≥ 60	6735/6724	Latin America, Argentina, Brazil, Chile, Colombia, Mexico, Peru, South Africa, the United States	III, 8, double-blind	55.0% (42.9, 64.7)†
Sahly 2021 (71)	mRNA-1273	2, 28 d, 100 µg	≥ 65	3626/3595	the United States	III, 99, observer-blinded	91.5% (83.2, 95.7)
Thomas 2021 (73)	BNT162b2	2, 21 d, 30 µg	≥ 55	8194/8208	the United States, Argentina, Brazil, Germany, South Africa, Turkey	II/III, 152, observer-blinded	90.9% (86.3, 94.2)†

Vp, viral particles. The vaccine efficacy (with 95% confidence intervals) in this table was based on what was reported in the original literature. †Indicated that the vaccine efficacy in the original literature was calculated on person-years, the total time for the given endpoint across all participants at risk within each group. *Indicated that the vaccine efficacy in the original literature was calculated based on symptomatic cases of COVID-19.

**Table 3:** Characteristics of included studies on the immunogenicity of COVID-19 vaccines.

Study	Vaccine	Administration (dosage, no. of doses)	Age range	No. of participants (n/N)	Country	Immunoassay days (D 1, D 2)	Study types (phase, no. of centers, blinding)	Estimated seroconversion rate (95% CI)
Asano 2022§ (49)	ChAdOx1-S-(AZD1222)	5 × 10^10^ VP, 2	≥ 56	49/86	Japan	D 56, D 28	I/II, 5, double-blind	56.90% (46.42, 67.07)
Bueno 2021† (51)	CoronaVac	3 µg, 2	≥ 60	−/27	Chile	D 42, D 28	III, 8, observer-blinded	−
Chu 2021 (52)	mRNA-1273	50 µg/100 µg, 2	≥ 55	140/140	the United States	D 56, D 28	II, 8, observer-blinded	99.82% (98.45, 99.84)
Ella 2021 (53)	BBV152 vaccine	6 µg, 2	≥ 60	−/52	Indian	D 56, D 28	III, 25, double-blind	−
Formica 2021 (55)	NVX-CoV2373	5 µg/25 µg, 2	≥ 60	51/52	the United States, Australia	D 35, D 14	II, 17, observer-blinded	97.25% (91.20, 99.90)
Guo 2021† (56)	WIBP COVID-19 vaccine	2.5 µg/5 µg/10 µg, 3	≥ 60	219/247	China	D 112, D 28	I/II, 2, double-blind	88.51% (84.25, 92.17)
Hsieh 2021 (60)	MVC-COV1901	15 µg, 2	≥ 65	220/221	China	D 56, D 28	II, 11, double-blind	99.34% (97.86, 99.98)
Li 2021† (62)	BNT162b1	10 µg/30 µg, 2	≥ 65	43/46	China	D 42, D 21	I, 1, double-blind	92.59% (83.47, 98.25)
Liu 2021 (63)	KCONVAC	5 µg/10 µg, 3	≥ 60	176/179	China	D 112, D 28	II, 3, double-blind	98.07% (95.55, 99.56)
Logunov 2021 (64)	Gam-COVID-Vac	10 × 10^11^ VP, 2	> 60	7/7	Russia	D 42, D 21	III, 25, double-blind	96.77% (74.69, 97.28)
Masuda 2022 (65)	mRNA-1273	100 mg, 2	≥ 65	49/49	Japan	D 56, D 28	I/II, 2, observer-blinded	99.50% (95.67, 99.54)
Meng 2021 (66)	Recombinant COVID-19 vaccine (Sf9 cells)	40 µg, 3	≥ 60	86/116	China	D 70/72, D 28	I/II, 1, double-blind	73.93% (65.63, 81.45)
Richmond 2021 (68)	SCB-2019	3 µg/9 µg/30 µg, 2	≥ 55	23/24	Australia	D 49, D 28	I, 1, double-blind	94.16% (81.85, 99.77)
Sadoff 2021 (69)	Ad26.COV2.S	5 × 10^10^/1 × 10^11^ VP, 1	≥ 65	92/100	Belgium, the United States	D 28, D 28	I/II a, 12, double-blind	91.59% (85.42, 96.18)
Shu 2021 (72)	Recombinant SARS-CoV-2 Fusion Protein Vaccine (V-01)	10 µg/25 µg, 2	≥ 60	225/232	China	D 49, D 28	II, 1, double-blind	96.78% (94.14, 98.66)
Wu 2021* (75)	CoronaVac	3 µg/6 µg, 2	≥ 60	239/243	China	D 56, D 28	I/II, 1, double-blind	98.16% (96.10, 99.46)
Xia 2021 (76)	BBIBP-CorV	2 µg/4 µg/8 µg, 2	≥ 60	70/70	China	D 42, D 14	I, 1, double-blind	99.65% (96.94, 99.68)
Zeng 2022* (22)	CoronaVac	1.5 µg/3 µg/6 µg, 3	≥ 60	83/85	China	D 268, D 28	II, 1, double-blind	97.12% (92.57, 99.58)
Zhang 2021 (77)	Recombinant SARS-CoV-2 Fusion Protein Vaccine (V-01)	10 µg/25 µg/50 µg, 2	≥ 60	−/68	China	D 56, D 28	I, 1, double-blind	−
Zhu 2020 (78)	Ad5-nCoV	5 × 10^10^/1 × 10^11^ VP, 1	≥ 55	14/51	China	D 28, D 28	II, 1, double-blind	27.88% (16.65, 40.73)
Zhu 2021§ (79)	Ad5-nCoV	5 × 10^10^/1 × 10^11^ VP, 2	≥ 56	183/198	China	D 84, D 28	II b, 1, double-blind	92.21 (88.10, 95.52)

Vp, viral particles; nAb, neutralizing antibody; n, the number of participants in vaccination group with seroconversion; N, total number of per-protocol set in vaccination group to assessed SARS-CoV-2-specific nAb. D 1 and D 2 refer to the same day; D 1, days after first vaccination; D 2, days after the last vaccination. (The day of the first dose of the vaccine is Day 0). *Two articles are from the same trial. †SARS-CoV-2-specific neutralizing antibody titer was assessed based on the variant virus strain. §SARS-CoV-2-specific neutralizing antibody titer was assessed based on the pseudovirus.

### Efficacy of COVID-19 vaccines

**Figure 2:**
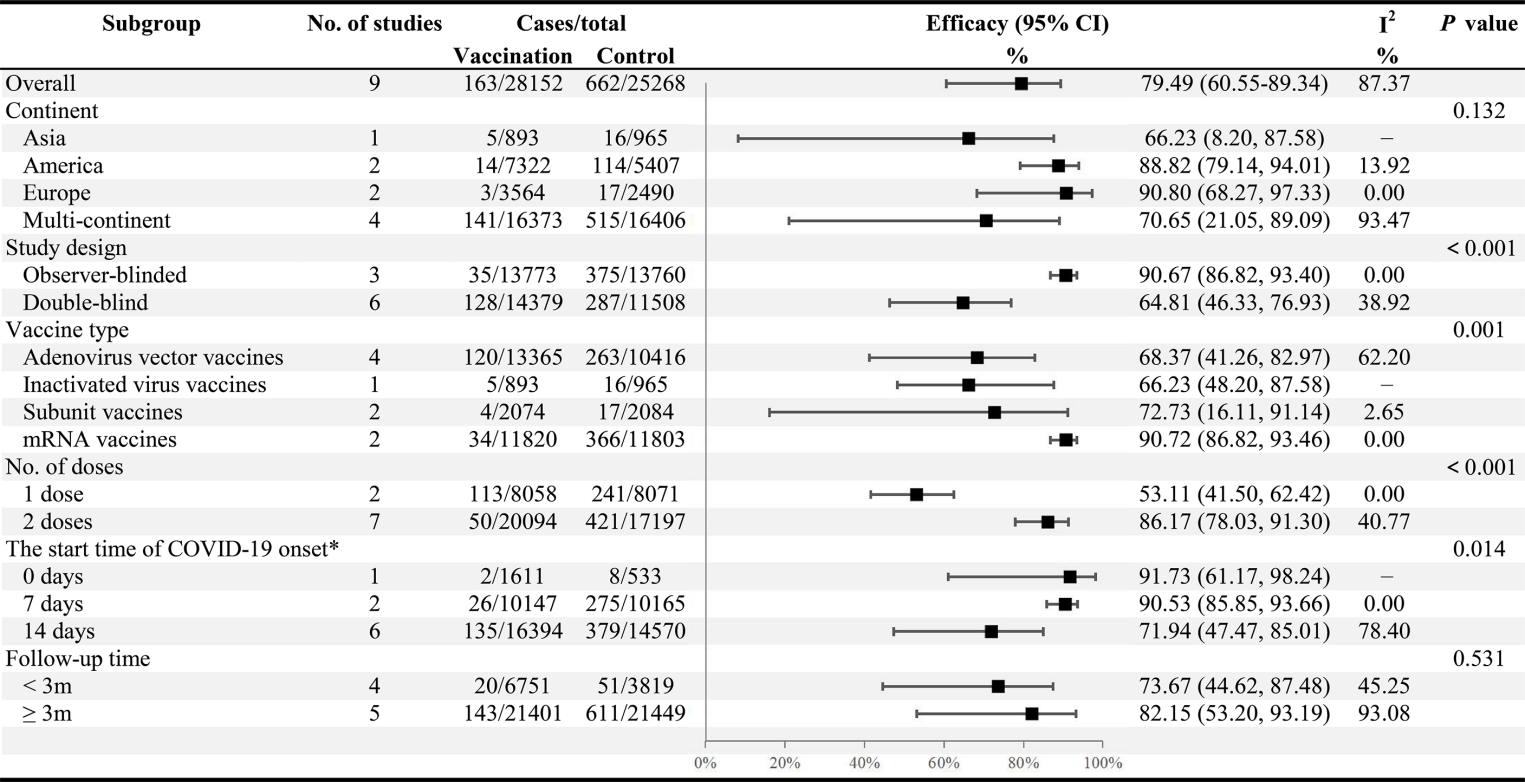
Subgroup analysis of the COVID-19 vaccine efficacy across variables. *Indicated the start time for the accrual of COVID-19 cases after the last vaccination (the day of the last vaccination is Day 0).

Nine studies were included to evaluate the efficacy of COVID-19 vaccines (50, 53, 54, 57, 59, 64, 70, 71, 73), comparing SARS-CoV-2 infection in 28152 vaccinated older adults with 25268 who received a placebo. Four studies focus on adenovirus vector vaccines (54, 57, 64, 70), two on subunit vaccines (50, 59), two on mRNA vaccines (71, 73), and one on inactivated virus vaccines (53) (**Table 1**). As shown in **Table 2**, five studies reported efficacy above 80% (54, 59, 64, 71, 73), while the remaining four showed efficacy between 50% and 70%. Collectively, we observed an efficacy of 79.49% (95% CI: 60.55−89.34) for COVID-19 vaccines (**Figure 2**). Optimistically, COVID-19 vaccines provided a marked protection rate against symptomatic SARS-CoV-2 infection (53, 59, 64, 70), especially reducing the risk of severe cases (57, 70, 71), with a combined efficacy of 72.26% (37.56−87.68) and 87.01% (50.80−96.57), respectively; however, the evidence was limited due to insufficient studies (**Figures S1B, C**). Besides, COVID-19 vaccines also showed promising efficacy, in different age groups, including aged ≥ 60 years (85.34%, 95% CI: 71.65−92.42) (50, 54, 59, 71) and aged ≥ 75 years (96.53%, 82.34−99.32) (71, 73) (**Figure S2A**). According to the GRADE system, the certainty of the evidence for vaccine efficacy was moderate (**Table S6**).

There was substantial heterogeneity in the summary efficacy (I^2^ = 87.37%), as presented in the Galbraith plot (**Figure S5A**). Consequently, subgroup and meta-regression analyses were performed to explore the potential heterogeneity. In the subgroup analysis (**Figure 2**), the pooled vaccine efficacy (VE) varied across study design (*p* < 0.001), vaccine types (*p* = 0.001), the number of vaccination doses (*p* < 0.001), and the start time of COVID-19 onset (*p* = 0.014), all of which may have contributed to significant heterogeneity. Among the four types of vaccines, mRNA vaccines reported the highest efficacy (90.72%, 95% CI: 86.82−93.46), followed by subunit vaccines (72.73%, 16.11−91.14). Adenovirus vector vaccines (68.37%, 41.26−82.97) and inactivated virus vaccines (66.23%, 48.20−87.58) exhibited similar and inferior combined efficacy (**Figure 2**). Vaccine efficacy was higher in two-dose vaccine recipients (86.17%, 78.03−91.30) than in one-dose vaccination (53.11%, 41.50−62.42). In addition, the majority of studies were double-blind (6/9) with an estimated efficacy of 64.81% (46.33−76.93), considerably lower than the efficacy of the observer-blinded trials (90.67%, 86.82−93.40). However, the continent and follow-up time did not contribute to significant heterogeneity (**Figure 2**).

In univariate meta-regression analysis, we likewise found that vaccine type and the number of doses were crucial factors influencing VE, suggesting the sources of heterogeneity (**Table 4**).

**Table 4:** The results of univariate and multivariate meta-regression analyses on vaccine efficacy.

Variables		Coefficient	95% CI	Std. Err	*P* value
**Univariate Analysis**					
** Continent**	Europe	ref	ref	ref	ref
	Asia	1.2804	(−1.3680, 3.9287)	1.3512	0.343
	America	0.2602	(−1.9545, 2.4749)	1.1299	0.818
	Multi-continent	1.1368	(−0.8464, 3.1200)	1.0118	0.261
** Study design**	Observer-blinded	ref	ref	ref	ref
	Double-blind	−1.4338	(−1.9541, −0.9135)	0.2655	< 0.001
** Vaccine type**	Adenovirus vector vaccines	ref	ref	ref	ref
	Subunit vaccines	−0.2622	(−1.5704, 1.0460)	0.6675	0.694
	Inactivated virus vaccines	−0.0212	(−1.3167, 1.2744)	0.6610	0.974
	mRNA vaccines	−1.3200	(−2.0974, −0.5425)	0.3967	0.001
** No. of doses**	1 dose	ref	ref	ref	ref
	2 doses	−1.2718	(−1.9105, −0.6331)	0.3259	< 0.001
The start time of COVID-19 onset*	14 days	ref	ref	ref	ref
	7 days	−1.0557	(−2.3472, 0.2357)	0.6589	0.109
	0 day	−1.2230	(−3.2895, 0.8435)	1.0543	0.246
** Follow-up time**	< 3m	ref	ref	ref	ref
	≥ 3m	−0.3166	(−1.7362, 1.1030)	0.7243	0.662
**Multivariate Analysis**					
** Study design**	Observer-blinded	ref	ref	ref	ref
	Double-blind	−1.2627	(−3.7047, 1.1792)	1.2459	0.311
** Vaccine type**	Adenovirus vector vaccines	ref	ref	ref	ref
	Subunit vaccines	1.0372	(−0.5194, 2.5939)	0.7942	0.192
	Inactivated virus vaccines	0.8841	(−0.4292, 2.1973)	0.6700	0.187
	mRNA vaccines	0.8553	(−1.7544, 3.4650)	1.3315	0.521
** No. of doses**	1 dose	ref	ref	ref	ref
	2 doses	−1.2123	(−2.0918, −0.3328)	0.4487	0.007

*Indicated the start time for the accrual of COVID-19 cases after the last vaccination (the day of the last vaccination is Day 0).

The sensitivity analysis showed that none of the nine studies was likely to have an inordinate influence on the reported VE estimates (**Figure S4A**). Egger’s test (*p* = 0.623) indicated a low likelihood of publication bias, although the funnel plot revealed a slight asymmetry (**Figure S3A**). The trim-and-fill analysis implied that the impact of non-published studies on the meta-results was minimal (**Figure S3A**).

### Immunogenicity of the COVID-19 vaccines

**Figure 3:**
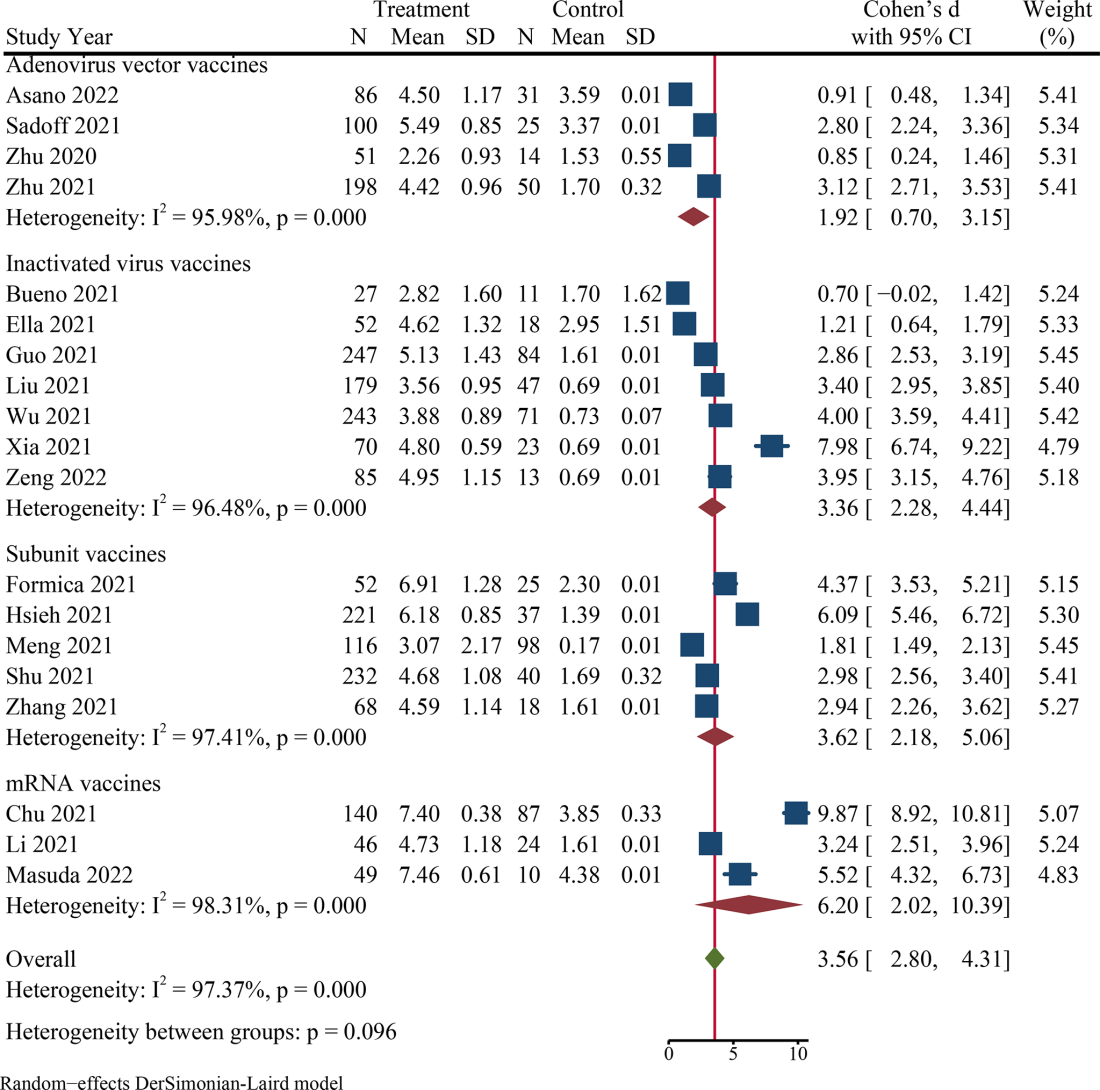
The forest plot of GMT values of log-transformed neutralizing antibody.

**Figure 4:**
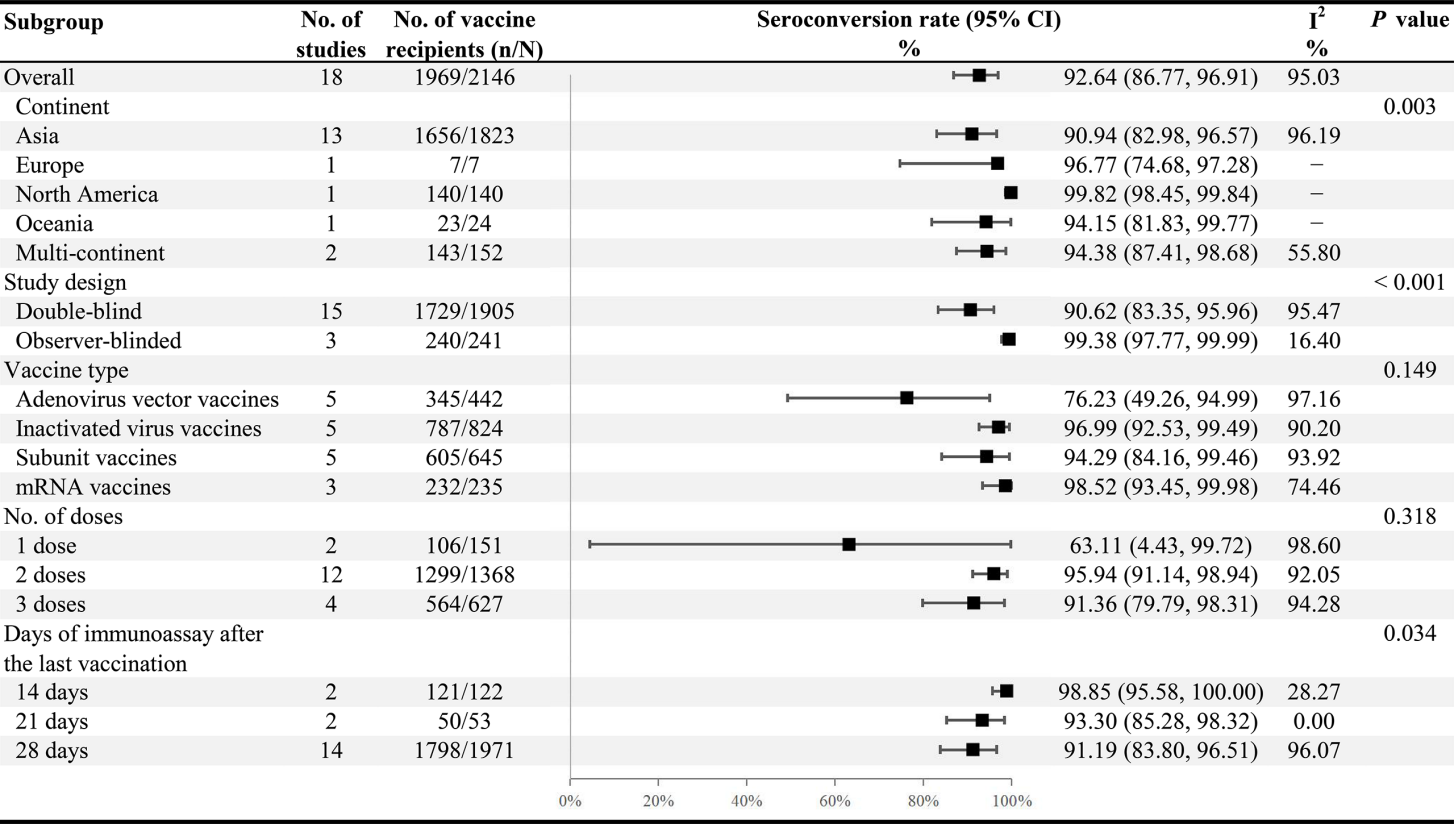
Subgroup analysis of the neutralizing antibody seroconversion rates across variables. N, the total number of vaccinated participants assessing neutralizing antibodies for SARS-CoV-2; n, the number of participants with seroconversion.

Twenty-one published studies described data on immunogenicity, including 7 studies of inactivated virus vaccines (22, 51, 53, 56, 63, 75, 76), 6 studies of subunit vaccines (55, 60, 66, 68, 72, 77), 5 studies of adenovirus vector vaccines (49, 64, 69, 78, 79), and 3 studies of mRNA vaccines (52, 62, 65) (**Table 1**). Thirteen studies (49, 55, 60, 62–66, 68, 69, 72, 78, 79) reported the seroconversion rate of spike-specific IgG or RBD-specific IgG. Among the 21 included studies, most immunoassay days were 28th after the last vaccination (n = 17), and a few were day 14 (n = 2) (55, 76) or day 21 (n = 2) (62, 64). It is clear from **Table 3** that most studies had a neutralizing antibody seroconversion rate above 90%, with only four studies below 90% (49, 56, 66, 78), the lowest of which was the adenovirus vector vaccines at a mere 27.88% (95% CI: 16.65−40.73) (78). After vaccination, the pooled neutralizing antibody seroconversion rate and the SMD value of neutralizing antibody GMT were 92.64% (95% CI: 86.77−96.91) and 3.56 (95% CI: 2.80−4.31), respectively (**Figure 3** and **Figure 4**). Surprisingly, we also observed a high seroconversion rate (96.85%, 91.11−99.72) and GMT value (SMD 4.39, 2.64−6.13) of neutralizing antibodies in people aged ≥ 65 years after vaccination (**Figures S2B, C**). The quality of evidence for neutralizing antibody seroconversion rate was low according to the GRADE system (**Table S6**).

Significant heterogeneity was noted among studies in seroconversion rate (I^2^ = 95.03%), and it was also shown in the Galbraith plot (**Figure S5B**). Subgroup analysis of neutralizing antibody seroconversion rate indicated that continent (*p* = 0.003), study design (*p* < 0.001), and the days of immunoassay (*p* = 0.034) made a substantial contribution to heterogeneity (**Figure 4**). However, vaccine type and the number of doses failed to explain the sources of heterogeneity. Among different continents, most of the studies were conducted in Asia (13/21), with a summary seroconversion rate of 90.94% (95% CI: 82.98−96.57). Concerning the study design, the combined seroconversion rate was 99.38% (97.77−99.99) in observer-blinded trials (52, 55, 65) compared to 90.62% (83.35−95.96) in double-blind trials (22, 49, 56, 60, 62–64, 66, 68, 69, 72, 75, 76, 78, 79). Of all vaccines, the highest seroconversion rate was observed in mRNA vaccines (98.52%, 93.45−99.98), and the lowest was in adenovirus vector vaccines (76.23%, 49.26−94.99). Most notably, the seroconversion rate was significantly lower in persons receiving one dose of vaccines (63.11%, 4.43−99.72) than in those receiving two or more doses (> 90%) (**Figure 4**). We found similar pooled results in neutralizing antibody GMT, which were the highest for mRNA vaccines (SMD 6.20, 95% CI: 2.02−10.39) but the lowest for adenoviral vector vaccines (SMD 1.92, 0.70−3.15) (**Figure 3**). Additionally, COVID-19 vaccines, in particular the mRNA vaccines, had an excellent ability to induce both spike-specific IgG and RBD-specific IgG, similar to the results of the neutralizing antibody response. The combined seroconversion rates showed 98.54% (95% CI: 97.13−99.49) for spike-specific IgG and 94.72% (88.35−98.68) for RBD-specific IgG (**Table S2**).

Univariate meta-regression indicated that vaccine type and the number of vaccination doses affected the heterogeneity of the neutralizing antibody seroconversion rate. In contrast, the continent, study design, and the days of immunoassay did not significantly contribute to heterogeneity (**Table S4**).

Sensitivity analysis on neutralizing antibody seroconversion rate was presented in **Figure S4B**, suggesting that no single included study remarkably impacted the reported immunogenicity results. Egger’s test found no evident publication bias (*p* = 0.773). The slightly asymmetric funnel plot and trim-and-fill analysis showed little effect on publication bias (**Figure S3B**).

### Safety of COVID-19 vaccines

Twenty-five studies were eligible to pool the risk of adverse events (AEs), including total AEs, systemic AEs, local AEs, and other specific AEs. Among all included studies for safety analyses, five types of vaccines were analyzed: adenovirus vector vaccines (n = 6) (49, 54, 64, 67, 69, 79), inactivated virus vaccines (n = 6) (22, 51, 56, 63, 75, 76), subunit vaccines (n = 6) (55, 60, 66, 68, 72, 77), mRNA vaccines (n = 6) (52, 58, 62, 65, 71, 74), and DNA vaccines (n = 1) (61) (**Table 1**). Approximately one-third of the included studies were observer-blinded (8/25) (51, 52, 55, 58, 65, 67, 71, 74), and the remainder were double-blind (17/25) (**Table S1**). As presented in **Table 5**, COVID-19 vaccines were associated with some included AEs compared to the control group (RR range from 1.74 to 3.82), except for vomiting or nausea and diarrhea. The combined risk ratios of total, systemic and local adverse events were 1.59 (95% CI: 1.38−1.83), 1.55 (1.30−1.85), and 3.42 (2.74−4.28), respectively. We observed that mRNA vaccination resulted in the highest risk of AEs (RR range from 1.74 to 7.22), except for redness (5.01, 2.15−11.65), diarrhea (0.41, 0.08−2.12), and arthralgia (2.00, 1.17−3.43). The most frequent local and systemic AEs of mRNA vaccines were swelling (7.22, 3.41−15.28) and fever (4.31, 1.18−15.72), respectively. In contrast, vaccines with favorable safety profiles were mainly inactivated virus vaccines (RR range from 0.40 to 1.47, *p* > 0.1) and DNA vaccines (n = 1 study, RR range from 0.36 to 1.99, *p* > 0.1), with no higher risk of AEs observed in the post-vaccination population. **Table S6** shows that the certainty of the evidence for both systemic and local AEs was low.

**Table 5:** Incidence of adverse events among the vaccination versus the control group.

Adverse events	Vaccine type	No. of Studies	Reactions/total		RR (95% CI)	Heterogeneity I^2^ (%)	Test of effect size (*p* value)
			Vaccination	Control			
Total adverse events	Adenovirus vector vaccines	3	513/1030	67/260	1.84 (1.50, 2.27)	0.00	< 0.001
	DNA vaccines	1	31/2678	41/2664	0.75 (0.37, 1.54)	55.06	0.441
	Inactivated virus vaccines	5	147/1472	37/351	0.94 (0.63, 1.41)	17.33	0.773
	Subunit vaccines	3	108/1644	24/352	0.91 (0.60, 1.37)	0.00	0.641
	mRNA vaccines	5	6821/8070	2975/7658	2.08 (1.77, 2.45)	87.79*	< 0.001
	Overall	17	7620/14894	3144/11285	1.59 (1.38, 1.83)	80.33*	< 0.001
Systemic adverse events (any)	Adenovirus vector vaccines	5	1051/2594	276/1013	1.67 (1.37, 2.02)	40.84*	< 0.001
	Inactivated virus vaccines	3	57/1144	20/280	0.67 (0.41, 1.10)	0.00	0.113
	Subunit vaccines	5	717/3317	113/724	1.41 (1.17, 1.71)	7.74	< 0.001
	mRNA vaccines	3	4715/7899	2540/7620	1.77 (1.23, 2.55)	98.05*	0.002
	Overall	16	6540/14954	2949/9637	1.55 (1.30, 1.85)	89.86*	< 0.001
Local adverse events (any)	Adenovirus vector vaccines	6	1118/3623	146/1353	2.75 (2.14, 3.55)	44.44*	< 0.001
	Inactivated virus vaccines	3	68/1144	14/280	1.47 (0.50, 4.35)	55.48*	0.488
	Subunit vaccines	5	907/3315	48/714	3.02 (1.42, 6.40)	80.86*	0.004
	mRNA vaccines	3	6234/7899	1084/7620	6.08 (4.83, 7.64)	83.32*	< 0.001
	Overall	17	8327/15981	1292/9967	3.42 (2.74, 4.28)	85.44*	< 0.001
Pain	Adenovirus vector vaccines	4	661/2286	81/934	3.28 (2.46, 4.38)	19.70*	< 0.001
	DNA vaccines	1	7/2678	11/2664	0.64 (0.25, 1.64)	1.00	0.351
	Inactivated virus vaccines	6	85/1522	18/371	1.08 (0.65, 1.81)	0.00	0.766
	Subunit vaccines	6	732/3104	32/680	3.88 (1.60, 9.43)	78.26*	0.003
	mRNA vaccines	6	6412/8232	936/7716	6.67 (5.81, 7.65)	27.04	< 0.001
	Overall	23	7897/17822	1078/12365	3.82 (3.01, 4.86)	77.14*	< 0.001
Redness	Adenovirus vector vaccines	4	49/2190	6/902	1.87 (0.66, 5.27)	26.34	0.240
	DNA vaccines	1	2/869	1/863	1.99 (0.18, 21.86)	−	0.575
	Inactivated virus vaccines	4	5/880	2/213	0.48 (0.12, 1.97)	0.00	0.307
	Subunit vaccines	3	60/2171	0/427	5.25 (1.48, 18.63)	0.00	0.010
	mRNA vaccines	6	416/8034	39/7649	5.01 (2.15, 11.65)	64.76*	< 0.001
	Overall	18	538/14188	48/10115	2.87 (1.58, 5.23)	57.51*	0.001
Swelling	Adenovirus vector vaccines	3	16/818	4/213	0.88 (0.33, 2.35)	0.00	0.797
	DNA vaccines	1	2/1793	6/1786	0.36 (0.07, 1.99)	0.00	0.243
	Inactivated virus vaccines	3	4/969	1/238	0.59 (0.13, 2.70)	0.00	0.496
	Subunit vaccines	3	103/2219	0/484	5.58 (1.73, 17.96)	0.00	0.004
	mRNA vaccines	6	636/8112	38/7658	7.22 (3.41, 15.28)	56.36*	< 0.001
	Overall	16	765/13911	49/10395	2.71 (1.41, 5.19)	65.02*	0.003
Fever	Adenovirus vector vaccines	5	56/3315	5/1274	1.91 (0.83, 4.42)	0.00	0.130
	DNA vaccines	1	3/1754	3/1741	0.99 (0.20, 4.91)	0.00	0.993
	Inactivated virus vaccines	4	21/1216	5/304	0.84 (0.32, 2.15)	0.00	0.710
	Subunit vaccines	5	40/2983	5/647	1.37 (0.61, 3.06)	0.00	0.444
	mRNA vaccines	6	449/8152	13/7691	4.31 (1.18, 15.72)	77.42*	0.027
	Overall	21	572/17442	31/11665	2.20 (1.20, 4.04)	60.35*	0.011
Headache	Adenovirus vector vaccines	5	497/3315	119/1274	1.75 (1.45, 2.10)	0.00	< 0.001
	DNA vaccines	1	7/2678	5/2664	1.35 (0.40, 4.53)	0.00	0.626
	Inactivated virus vaccines	4	17/1070	4/237	0.80 (0.33, 1.94)	0.00	0.626
	Subunit vaccines	6	249/3052	53/660	1.07 (0.81, 1.41)	0.00	0.646
	mRNA vaccines	6	2846/8188	1415/7700	2.05 (1.35, 3.11)	91.51*	0.001
	Overall	22	3629/18347	1597/12551	1.56 (1.23, 1.96)	79.24*	< 0.001
Fatigue	Adenovirus vector vaccines	5	533/3315	146/1274	1.54 (1.30, 1.82)	0.00	< 0.001
	DNA vaccines	1	3/1809	5/1801	0.60 (0.14, 2.55)	0.00	0.489
	Inactivated virus vaccines	5	22/1322	6/321	0.69 (0.28, 1.68)	0.00	0.409
	Subunit vaccines	6	356/3106	59/678	1.37 (1.01, 1.87)	16.74	0.046
	mRNA vaccines	6	3674/8188	1620/7700	1.90 (1.29, 2.81)	93.51*	0.001
	Overall	23	4588/17740	1836/11774	1.57 (1.24, 1.98)	84.54*	< 0.001
Chill	Adenovirus vector vaccines	2	149/1564	33/753	2.07 (1.12, 3.82)	41.19	0.020
	mRNA vaccines	6	1450/8210	307/7708	2.52 (1.10, 5.76)	93.11*	0.028
	Overall	8	1599/9774	340/8461	2.47 (1.27, 4.77)	91.57*	0.007
Vomiting or Nausea	Adenovirus vector vaccines	5	115/3115	43/1224	1.12 (0.79, 1.57)	0.00	0.525
	Inactivated virus vaccines	3	7/653	4/141	0.40 (0.08, 1.88)	30.70	0.243
	Subunit vaccines	5	83/3034	11/660	1.30 (0.70, 2.42)	0.00	0.399
	mRNA vaccines	3	663/7936	306/7615	1.74 (0.79, 3.81)	91.55*	0.169
	Overall	16	868/14738	364/9640	1.26 (0.84, 1.88)	73.31*	0.261
Diarrhea	Adenovirus vector vaccines	1	8/1029	1/340	2.64 (0.33, 21.06)	−	0.359
	Inactivated virus vaccines	4	10/1066	5/274	0.51 (0.18, 1.48)	0.00	0.215
	Subunit vaccines	4	93/2294	14/420	0.78 (0.18, 3.47)	60.94*	0.747
	mRNA vaccines	1	5/144	2/36	0.41 (0.08, 2.12)	14.69	0.289
	Overall	10	116/4533	22/1070	0.73 (0.36, 1.47)	28.17	0.372
Arthralgia	Adenovirus vector vaccines	2	6/1229	1/390	1.30 (0.22, 7.67)	0.00	0.770
	Inactivated virus vaccines	3	4/374	0/128	1.13 (0.23, 5.45)	0.00	0.880
	Subunit vaccines	3	66/1499	5/400	3.36 (1.47, 7.67)	0.00	0.004
	mRNA vaccines	5	2035/8188	874/7700	2.00 (1.17, 3.43)	91.77*	0.011
	Overall	13	2111/11290	880/8618	2.06 (1.33, 3.19)	84.68*	0.001
Malaise	Adenovirus vector vaccines	3	260/2593	72/1093	1.73 (1.35, 2.21)	0.00	< 0.001
	Subunit vaccines	1	89/686	17/222	1.79 (0.94, 3.39)	40.11	0.076
	mRNA vaccines	1	7/94	1/48	2.31 (0.40, 13.31)	0.00	0.350
	Overall	5	356/3373	90/1363	1.74 (1.40, 2.17)	0.00	< 0.001
Myalgia	Adenovirus vector vaccines	5	424/3315	92/1274	1.92 (1.40, 2.62)	21.21	< 0.001
	DNA vaccines	1	2/1793	5/1786	0.40 (0.08, 2.08)	0.00	0.278
	Inactivated virus vaccines	4	10/1070	6/237	0.44 (0.17, 1.17)	0.00	0.102
	Subunit vaccines	4	327/2581	26/562	2.19 (1.43, 3.34)	10.27	< 0.001
	mRNA vaccines	6	2666/8162	868/7676	2.26 (1.32, 3.85)	93.69*	0.003
	Overall	20	3429/16921	997/11543	1.90 (1.38, 2.60)	84.85*	< 0.001

*The test of heterogeneity: *p* < 0.1.

**Figure 5:**
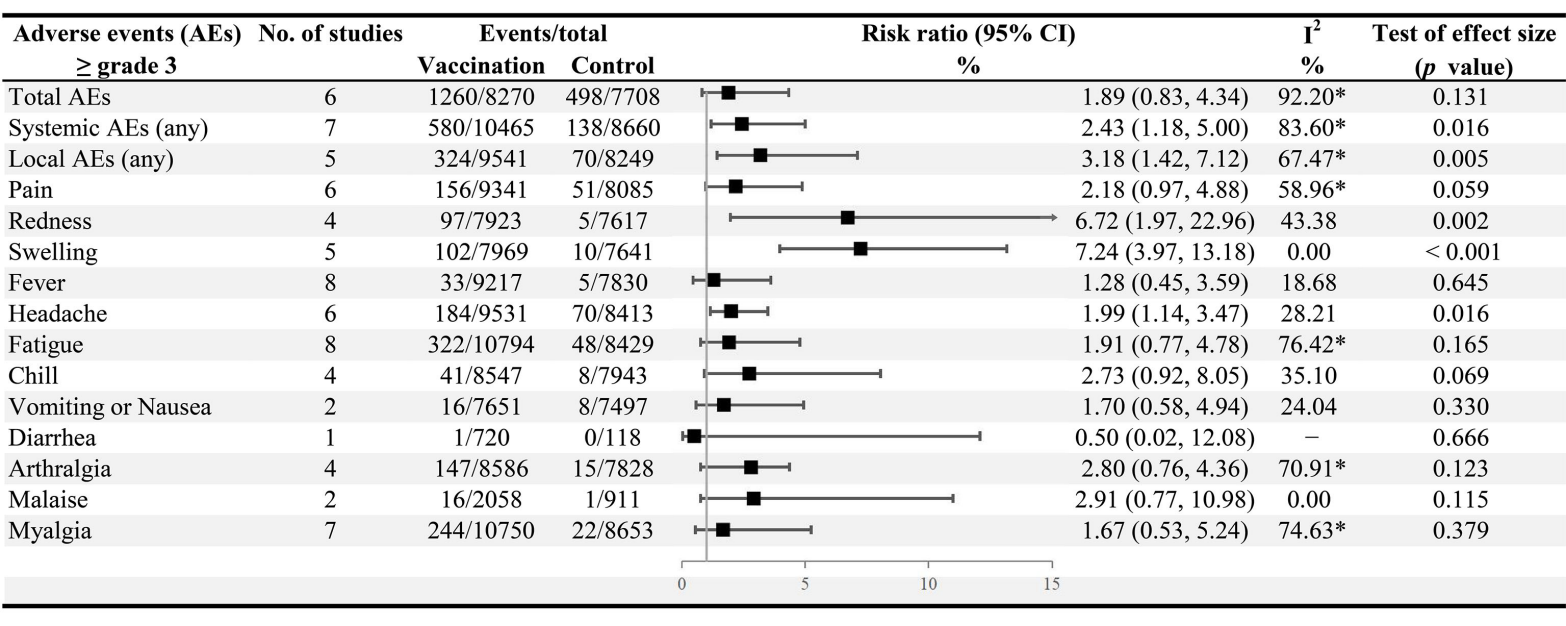
Incidence of grade ≥ 3 adverse events among the vaccination versus the control group. *The test of heterogeneity: *p* < 0.1.

For all grade ≥ 3 AEs included in the analysis, the risk of occurrence of local AEs (RR 3.18, 95% CI: 1.42−7.12), systemic AEs (2.43, 1.18−5.00), injection site redness (6.72, 1.97−22.96), swelling (7.24, 3.97−13.18), and the headache (1.99, 1.14−3.47) was markedly increased after vaccination (**Figure 5**). In addition, no statistical differences were observed in the incidence of total AEs (RR 1.09, 0.95−1.24), systemic AEs (1.14, 0.87−1.50), and local AEs (0.99, 0.88−1.12) between after the first and second doses of vaccination (**Figure S1H**). Moreover, compared to persons aged 55 years and older, people aged ≥ 65 years appeared to be more likely to experience AEs after vaccination (total AEs, RR 2.15, 95% CI: 1.78−2.59; systemic AEs, 1.73, 1.33−2.26; local AEs, 4.27, 3.45−5.30) (**Figures S2D–F**).

The heterogeneity of meta-analyses for total, system, and local AEs were relatively great (I^2^ = 80.33%, 89.86%, and 85.44%, respectively), and the Galbraith plots visually showed the heterogeneity (**Figures S5C–E**). In subgroup analysis of total AEs, different continents (*p* = 0.021), study design (*p* = 0.017), and vaccine types (*p* < 0.001) were the sources of the high heterogeneity. Of all vaccine types, only adenovirus vector vaccines (RR 1.84, 95% CI: 1.50−2.27) and mRNA vaccines (2.08, 1.77−2.45) were significantly associated with the total AEs. The double-blind trials (1.25, 0.89−1.75) had a lower incidence of total AEs to COVID-19 vaccines than the observer-blinded trials (1.95, 1.69−2.25) (**Table S3**).

Multivariate meta-regression analysis revealed that vaccine type and the number of injections significantly affected the occurrence of total AEs and contributed to considerable heterogeneity (**Table S5**).

The sensitivity analysis suggested that the results of the safety meta-analysis were relatively stable (**Figures S4 C–E**). No publication bias was observed for systemic and local AEs by visual examinations of the funnel plot and the Egger’s test (*p* = 0.729, *p* = 0.795, respectively) (**Figures S3D, E**). Moreover, the trim-and-fill analysis of total AEs confirmed the mild effect of potentially unpublished studies on the safety results (**Figure S3C**).

## Discussion

This systematic review and meta-analysis presents a comprehensive synthesis of current data on the efficacy, safety, and immunogenicity of COVID-19 vaccination in older adults based on 32 RCTs. We found that (1) vaccination was highly efficacious against SARS-CoV-2 infection in the elderly (79.49%), particularly providing a high level of protection against severe disease (87.01%) and for advanced age groups, e.g. over 75 years old (96.53%); (2) the mRNA vaccines appeared to be the most efficacious vaccines (90.72%), with the highest neutralizing antibody seroconversion rate (98.52%) and GMT (SMD, 6.20); (3) vaccine safety was acceptable for older adults and the risk of grade ≥ 3 AEs such as local redness, swelling, and the headache was significantly increased; (4) inactivated vaccines had excellent safety profile, with similar occurrence of any solicited AEs between the vaccination and control groups (p > 0.05), while the mRNA vaccines had the highest incidence of AEs (in addition to redness, diarrhea and arthralgia); (5) no significant differences of local and systemic AEs were observed between the first dose and the second dose (p > 0.05); (6) the type of vaccine was the primary predictor of the primary outcome, while the number of doses was the main predictor of efficacy and neutralizing antibody seroconversion rates. According to the GRADE system, the certainty of the evidence for vaccine efficacy was moderate, and for neutralizing antibody seroconversion rate and both systemic and local AEs were low.

With the rapid increase in clinical trials of COVID-19 vaccines, several meta-analyses and systematic reviews have been conducted on vaccine efficacy (VE), immunogenicity and safety in different age groups (37, 38, 80). The combined efficacy of COVID-19 vaccines found in this study (79.49%) was slightly lower than the pooled effectiveness based on real-world estimates in the elderly (83.8%) (37), and significantly lower than that in the children and adolescent population (96.09%) (80). Five (50, 53, 57, 59, 70) of the nine trials included for the efficacy analysis suggested lower efficacy in older adults than in younger adults, reflecting that of Cheng et al. (38) This discrepancy could be attributed to comorbidities and immunosenescence (typically defined as age-related structural and functional changes in the body’s innate and adaptive immune system) (78, 81). Older adults thus have poorer immune responses to COVID-19 vaccines than younger ones (32, 82). It likewise explains that serum neutralizing and binding antibodies decreased with age in the elderly cohort and were significantly lower in those ≥ 80 years old (83). Interestingly, our further analyses did not seem to support this diminishing phenomenon, that is, vaccine efficacy (71, 73) and immune response were not lower in advanced older adults than in younger elderly. There are several factors that can likely explain this observation. Firstly, in general, the older the participants in the original study, the smaller the sample size of this population, which led, partly, to a small sample study effect. Secondly, the types of vaccines and the physical fitness of the individuals included in the various studies differed (e.g., some studies included individuals with stable comorbidities, and some included only perfectly healthy individuals), contributing to the significant variation in immune responses to vaccination. Most importantly, specific data for age stratification was unavailable, resulting in an inability to compare different age groups directly (e.g., ≥ 75 years vs. < 75 years).

We found that vaccine types were one of the main influencing factors for efficacy and immunogenicity outcomes. According to our evidence, adenovirus vector vaccines had the lowest neutralizing antibody seroconversion rate (76.23%), while mRNA vaccines showed the highest (98.52%). There is no doubt that mRNA vaccines were the most efficacious, which the efficacy analysis in this study and other literature have confirmed (38, 39). However, the estimated efficacy of inactivated and adenoviral vector vaccines was comparable and lower (66.23% versus 68.37%). Since only one trial was used for the efficacy analysis of inactivated vaccines, it cannot be convincingly concluded which vaccine was the worst. Previous meta-analyses based on all persons aged 18 years and older found that inactivated vaccines were likely to have the weakest protective effect (38, 39). Inactivated vaccines generally consist of intact virus or split segments, where the virus is killed, and its shell is preserved (84). They are unable to replicate in the recipients and cannot induce host cell interference (85), thus having a certain probability of failing to induce immune memory (86), which could theoretically explain our results. The mRNA vaccine is a nucleic acid sequence that, once injected into the human body, in addition to immunity activation by exogenous antigens produced by cells in the recipients, autologous cells expressing the antigens may also elicit a more robust cellular and humoral immune response (84, 87–89). Although our data suggest that mRNA vaccines had the relatively highest incidence of AEs of all vaccine types, they can be rapidly produced *in vitro* and have robust efficacy, making them desirable for a prompt response to SARS-CoV-2 (84, 90). Therefore, for regions with the widespread transmission of SARS-CoV-2 and high-level infection rates in the elderly, mRNA vaccines are valuable candidates for timely and effective interruption of COVID-19 transmission.

Furthermore, this present study showed a significant positive correlation between the number of doses and the efficacy and immunogenicity of COVID-19 vaccines. Participants who received one dose had only 53.11% efficacy and only 63.11% seroconversion rate of neutralizing antibodies, which were far inferior to those who received two or more doses. All one-dose studies were for adenovirus vector vaccines (Ad26.COV2.S and Ad5-nCoV), for which the basal immunization regimen is single-dose. However, further booster injections have been found to significantly boost the efficacy of adenovirus vector vaccines; therefore, booster injections (two doses) are currently recommended by the World Health Organization (WHO) for priority vaccination groups (e.g., elderly, health workers, individuals with comorbidities) (91, 92). In addition, a study has shown that about half of the population aged 80 years or older achieved optimal virus neutralization only after a second dose of the mRNA vaccine (BNT162b2) (83). For vaccines with a basic immunization of two doses (e.g., BNT162b2 and CoronaVac), booster vaccination also can considerably boost the prevention of critical illnesses and deaths (protection rate of more than 95%), especially for the elderly (93–95). Consequently, it is imperative to accelerate the completion of basic immunization and take specific measures to enhance vaccine responses in older adults, such as boosters.

In addition to age, vaccine types, and the number of doses, most studies have suggested that country and duration since vaccination also affected the efficacy and immunogenicity of COVID-19 vaccines (40, 41, 96–98). Nevertheless, we did not detect significant correlations between continent and efficacy or immune effects. Previous studies have suggested that the vaccine efficacy against SARS-CoV-2 infection and symptomatic COVID-19 diminished over time, but the protection rate against the severe disease was maintained at a high level (41, 96). A possible explanation for this might be weakened immunity resulting from the loss of vaccine-induced immunoprotection. Furthermore, decreased VE appeared to occur more in extraordinarily fragile older persons (98), dramatically raising concerns about the long-term efficacy of COVID-19 vaccines. In the past, decreased immunity to other infectious diseases was typically approached with booster vaccine doses (99), further confirmed in the COVID-19 vaccines (93–95). Because the median follow-up time from the last vaccination dose to confirmed positive infection in none of the included studies was long enough (ranging from 1 month to 6 months), we could not provide meaningful evidence of the duration of VE in older adults.

Since its initial discovery, the more transmissible and insidious Omicron has rapidly become the predominant variant prevalent worldwide, intensely threatening the neutralizing efficacy of current COVID-19 vaccines (100, 101). The vulnerable population, particularly those unvaccinated or with coexisting underlying diseases, represents an unprecedented challenge in addressing Omicron spread (102). Several studies have confirmed that COVID-19 vaccines remain an effective protective measure in interrupting Omicron transmission; simultaneously, the mRNA vaccines, notably homologous or heterologous boosters, are worthy candidates for priority consideration (42, 100, 102–106). There was limited or no data on VE against various variants, including Omicron, in the elderly population. Therefore, the range of issues caused by SARS-CoV-2 variants in the geriatric population and the efficacy and immunization effects of COVID-19 vaccines against them warrant further in-depth study.

Local AEs were more prevalent than systemic AEs after vaccination, and there was no conclusive evidence to attribute serious AEs exclusively to COVID-19 vaccines. Consistent with our results, mRNA vaccines had the highest incidence of AEs except for a few AEs (43). Moreover, the mRNA vaccines were more related to severe AEs than other vaccine types (44). Our evidence suggested that both inactivated and DNA vaccines had better safety than others; however, this finding should be considered cautious due to limited data on AEs with DNA vaccines. Theoretically, these discrepancies may be attributed to differences in the strength of the immune responses to various vaccines (86, 88, 89), as corroborated by this review’s efficacy and immunization results. DNA vaccines are generally considered a superior safety profile because DNA plasmids neither replicate nor induce vector-mediated immunological responses in the host (107). DNA vaccines also have the advantage of low production costs and easy storage. However, there are significant concerns about their long-term safety, such as whether the DNA in the vaccines will integrate into human DNA with harmful consequences (61, 107). The only DNA vaccine currently approved for emergency marketing (ZyCoV-D) is a unique vaccine that uses a needle-free injection system. This injection method is painless and also reduces side effects after vaccination (61).

We observed that the occurrence of AEs after vaccination in older adults varied by continent and vaccine type. It has been claimed that age and gender were also correlated with the risk of AEs (108, 109).The elderly and male population were more likely to report serious adverse events and deaths (108, 109). This inconsistency may be due to disparities in the immune response to the vaccines associated with immune potency (108, 110). Notably, subgroup and regression analyses suggested that blinding was essential in the pooled safety results. The risk ratios for adverse events may have been overstated due to the inclusion of observer-blinded studies, so our results should be interpreted cautiously. Furthermore, further evaluation was conducted to determine whether the number of doses was related to the risk of AEs. Although a direct comparison of the incidence of AEs after the first and second doses showed no significant difference, we found a statistically significant difference in the AEs after the second vaccination dose at a higher risk than the first dose in the multivariate regression. A meta-analysis of all adults reported that whether the risk of AEs differed after the first and second doses of vaccination may depend on the vaccine types (109).

Our meta-analyses suggested significant heterogeneity, even though subgroup and meta-regression analyses were conducted. This heterogeneity might be partly due to vaccine types, number of doses, and study design (blinding), geographic variations, differences in population characteristics between studies, but the remaining unexplained heterogeneity remained significant. In this present review, regression analysis revealed that the heterogeneity in efficacy, immunogenicity, and safety of COVID-19 vaccines was mainly due to vaccine types and the number of doses. This may be because of the different immune mechanisms of the different vaccine types (84–89). We also found that geographical differences such as continents contributed to the high heterogeneity in safety of COVID-19 vaccines. This may be explained by differences in the intensity of the immune response owing to racial differences. In addition, differences in the basic characteristics of the patients included in the original studies, such as age range, gender and underlying disease status, etc. may be a potential source of heterogeneity. However, these characteristics were not available for further analysis. Therefore, the generalizability of our findings is limited and the results should be interpreted with caution, taking into account specific regional and demographic characteristics.

Older adults are the most vulnerable of the susceptible population to SARS-CoV-2 infection. They have been observed to have the most elevated mortality rates and higher risk of sequelae (111, 112), especially in the unvaccinated population (49, 54, 59, 70, 72). Therefore, COVID-19 vaccination is crucial for this vulnerable population. This study is the first to integrate all of the RCTs literature on the efficacy, immunogenicity, and safety of the COVID-19 vaccines in older adults, providing robust information for national decisions on public health. We found that COVID-19 vaccines, particularly the mRNA vaccines, provided excellent protection against COVID-19 infection in the elderly. And the boosters significantly enhanced the efficacy of the vaccines. Therefore, it is reasonable to encourage older adults to receive COVID-19 vaccines as early as possible and to receive booster shots. Government and health authorities should promote the efficacy and safety of the COVID-19 vaccines in the elderly to reduce vaccine hesitation. This approach can be used to fill the current gap of low vaccination rates in the older adult population in some regions to achieve the overall epidemic control goal of reducing infection and mortality in the elderly (70). In addition, the mRNA vaccine is a valuable candidate for areas with widespread SARS-CoV-2 transmission and high infection rates in the elderly, but its relatively high incidence of adverse events should also be considered. Considering the urgency of promoting vaccination of older people, a comprehensive analysis of the factors interfering with vaccination is essential. Simultaneously, the assessment for older adults with uncontrolled underlying diseases is also urgently warranted on the agenda. In addition to vaccination, more effective measures for older adults are needed to reduce the severe clinical outcomes associated with COVID-19.

The strengths of this review lie in the inclusion of high-quality RCTs through a comprehensive literature search and the use of rigorous inclusion and exclusion procedures to obtain precise and reliable results. However, this review has several potential limitations. First, the number of studies used to combine the efficacy of each vaccine type is relatively small. Second, preprinted studies were not included in this review, which will probably be available after our cutoff date. Third, potentially eligible data was not included in this analysis. Despite our attempts to contact the authors to obtain specific data on older adults, they, unfortunately, did not respond to our invitation. Fourth, the age range of the older adults selected for this study was 55 years or older, which may not represent the general elderly population. Fifth, of the 32 included studies, 10 were observer-blinded, and 15 were assessed as high risk, which may have caused selection bias and overstated the estimates. Sixth, we observed substantial heterogeneity in vaccine efficacy, immunogenicity, and safety estimates in older adults across studies. Seventh, there were insufficient data to evaluate the duration of efficacy and immune effects after vaccination, and whether the vaccines result in long-term adverse events, thus necessitating long-term surveillance and study for the large-scale older population. Eighth, RCTs on emerging variant strains, heterologous sequential booster, homologous booster, and non-inferiority trials were not included because the number of studies was insufficient for meta-analysis. More high-quality, multi-center, large-sample studies and complete information are warranted to fill these gaps in older adults. Finally, as most studies did not present data on outcomes stratified by age in the older people, insufficient evidence was available to support the age-related changes in vaccine efficacy, immunogenicity and safety in the older cohorts.

In conclusion, COVID-19 vaccines showed favorable efficacy, immunogenicity and safety in the elderly. In particular, they provided a high protection rate against severe disease and for those advanced age groups, e.g., over 75 years old. The mRNA vaccines were the most efficacious for preventing SARS-CoV-2 infection in older adults but had the relatively poorest safety profile. The several AEs resulting from mRNA vaccines, and the serious adverse events (≥ grade 3), deserve concern and further research. Notably, the number of doses was remarkably and positively correlated with the efficacy and immunogenicity of COVID-19 vaccines; therefore, improved coverage of boosters to enhance the vaccine response in older people is warranted. Meanwhile, additional multi-center, large-sample clinical studies in the elderly are urgently required for a broader range of vaccine types, extended periods of follow-up, and SARS-CoV-2 variant strains.

## Data availability statement

The original contributions presented in the study are included in the article/**Supplementary Material**. Further inquiries can be directed to the corresponding authors.

## Author contributions

ZL proposed the view, designed and participated in the whole research process. ZL, SHL, FL, YFL, and YLL made contributions to the literature searching, screening, and data extracting. PP and SL were responsible for data checking and producing tables and graphs. ZL, SHL, and FL analyzed the data results and co-authored the manuscript. TL and LH provided guidance on the research and participated in the final paper modification. ZL, SHL, and FL contributed equally to the overall research. ZL and TL decided on the specifics of submitting the article for publication. All authors contributed to the article and approved the submitted version.

## Conflict of interest

The authors declare that the research was conducted in the absence of any commercial or financial relationships that could be construed as a potential conflict of interest.

## Publisher’s note

All claims expressed in this article are solely those of the authors and do not necessarily represent those of their affiliated organizations, or those of the publisher, the editors and the reviewers. Any product that may be evaluated in this article, or claim that may be made by its manufacturer, is not guaranteed or endorsed by the publisher.
