# Supplementary material for: Efficacy, immunogenicity and safety of COVID-19 vaccines in older adults: a systematic review and meta-analysis
Source: Front Immunol. 2022 Sep 13;13:965971. doi: 10.3389/fimmu.2022.965971 (PMC9513208; doi:10.3389/fimmu.2022.965971)
Supplement: Supplementary file 1 [file DataSheet_1.doc]

**Supplementary Appendix**

**Literature search strategy**

**Supplementary Figure S1.** The forest plot of meta-analysis.

**Supplementary Figure S2.** The forest plots for the specific populations (aged ≥ 60, ≥ 65 or ≥ 75 years).

**Supplementary Figure S3.** The funnel plots after trim-and-fill analysis.

**Supplementary Figure S4.** Sensitivity analysis results.

**Supplementary Figure S5.**The Galbraith plot of meta-analysis.

**Supplementary Figure S6.** The results of literature quality (risk of bias) assessment.

**Supplementary Table S1.**Characteristics of included studies on the safety of COVID-19 vaccines.

**Supplementary Table S2.** Combined seroconversion rates of spike-specific and RBD-specific IgG to different vaccine types.

**Supplementary Table S3.** Subgroup analysis of the total adverse events of COVID-19 vaccines across variables.

**Supplementary Table S4.**The results of univariate and multivariate meta-regression analyses on the neutralizing antibody seroconversion rates.

**Supplementary Table S5.** The results of univariate and multivariate meta-regression analyses on total adverse events of COVID-19 vaccines.

**Supplementary Table S6.** Evaluation of the quality of evidence using GRADE system for primary outcomes.

**Literature search strategy**

**Coverage: from the inception to April 9, 2022**

Database: PubMed

Result: 1719

Search strings: ("COVID-19"[MeSH Terms] OR "SARS-CoV-2"[MeSH Terms] OR COVID-19[Title/Abstract] OR COVID 19[Title/Abstract] OR SARS-CoV-2[Title/Abstract] OR SARS Coronavirus 2[Title/Abstract] OR Severe Acute Respiratory Syndrome Coronavirus 2[Title/Abstract] OR 2019-nCoV[Title/Abstract] OR 2019 nCoV[Title/Abstract] OR nCoV-19[Title/Abstract] OR nCoV 19[Title/Abstract] OR Coronavirus Disease-19[Title/Abstract] OR Coronavirus Disease 19[Title/Abstract] OR Coronavirus Disease 2019[Title/Abstract] OR Coronavirus Disease-2019 Virus[Title/Abstract] OR nCoV[Title/Abstract] OR Novel Coronavirus[Title/Abstract]) **AND** ("Vaccines"[MeSH Terms] OR "Vaccination"[MeSH Terms] OR "COVID-19 Vaccines"[MeSH Terms] OR vaccin*[Title/Abstract] OR immunis*[Title/Abstract] OR immuniz*[Title/Abstract]) **AND** (safety[All Fields] OR adverse event*[All Fields] OR adverse reaction*[All Fields] OR side effects[All Fields] OR tolerance[All Fields] OR tolerabilit*[All Fields] OR reactogenicity[All Fields] OR effectiveness[All Fields] OR efficacy[All Fields] OR immunogenicity[All Fields] OR outcome*[All Fields]) **AND** ("Aged"[Mesh] OR "Aged, 80 and over"[Mesh] OR "Frail Elderly"[Mesh] OR "older*"[All Fields] OR "older adult*"[All Fields] OR "olds"[All Fields] OR "elder*"[All Fields] OR "aging"[All Fields] OR "ageing"[All Fields] OR "aged"[All Fields] OR "senior*"[All Fields] OR "geriatr*"[All Fields] OR "gerontolog*"[All Fields] OR "late-life"[All Fields] OR "late life"[All Fields] OR "vulnerab*"[All Fields]) **AND** ("Randomized Controlled Trial"[Publication Type] OR "Controlled clinical trial"[Publication Type] OR "Clinical Trial"[Publication Type] OR "Clinical Trial, Phase I"[Publication Type] OR "Clinical Trial, Phase II"[Publication Type] OR "Clinical Trial, Phase III"[Publication Type] OR "Clinical Trial, Phase IV"[Publication Type] OR "Multicenter Study"[Publication Type] OR "Comparative Study"[Publication Type] OR "Double-Blind Method"[Mesh] OR "Single-Blind Method"[Mesh] OR "Random Allocation"[Mesh] OR "Placebos"[Mesh] OR random*[Title/Abstract] OR "randomized controlled trial*"[All Fields] OR RCT[Title/Abstract] OR randomized trial*[Title/Abstract] OR controlled trial*[Title/Abstract] OR controlled stud*[Title/Abstract] OR clinical trial*[Title/Abstract] OR clinical stud*[Title/Abstract] OR controlled clinical stud*[Title/Abstract] OR controlled clinical trial*[Title/Abstract] OR single-blind*[Title/Abstract] OR double-blind*[Title/Abstract] OR treble-blind*[Title/Abstract] OR triple-blind*[Title/Abstract] OR cross-over*[Title/Abstract] OR placebo*[Title/Abstract] OR factorial*[Title/Abstract] OR assign*[Title/Abstract] OR allocat*[Title/Abstract] OR control*[Title/Abstract] OR trial*[Title/Abstract] OR volunteer*[Title/Abstract])

Database: EMBASE

Result: 2092

Search strings: ('coronavirus disease 2019'/exp OR 'Severe acute respiratory syndrome coronavirus 2'/exp OR 'COVID-19':ab,ti OR 'COVID 19':ab,ti OR 'SARS-CoV-2':ab,ti OR 'SARS Coronavirus 2':ab,ti OR 'Severe Acute Respiratory Syndrome Coronavirus 2':ab,ti OR '2019-nCoV':ab,ti OR '2019 nCoV':ab,ti OR 'nCoV-19':ab,ti OR 'nCoV 19':ab,ti OR 'Coronavirus Disease-19':ab,ti OR 'Coronavirus Disease 19':ab,ti OR 'Coronavirus Disease 2019':ab,ti OR 'Coronavirus Disease-2019 Virus':ab,ti OR 'nCoV':ab,ti OR 'Novel Coronavirus':ab,ti) **AND** ('vaccine'/exp OR 'SARS-CoV-2 vaccine'/exp OR 'vaccination'/exp OR 'vaccin*':ab,ti OR 'immunis*':ab,ti OR 'immuniz*':ab,ti) **AND** ('safety' OR 'adverse event*' OR 'adverse reaction*' OR 'side effects' OR 'tolerance' OR 'tolerabilit*' OR 'reactogenicity' OR 'effectiveness' OR 'efficacy' OR 'immunogenicity' OR 'outcome*') **AND** ('aged'/exp OR 'frail elderly'/exp OR 'aging'/exp OR 'gerontopsychiatry'/exp OR 'older*' OR 'older adult*' OR 'olds' OR 'elder*' OR 'aging' OR 'ageing' OR 'aged' OR 'senior*' OR 'geriatr*' OR 'gerontolog*' OR 'late-life' OR 'late life' OR 'vulnerab*') **AND** ('randomized controlled trial'/exp OR 'controlled clinical trial'/exp OR 'clinical trial'/exp OR 'phase 1 clinical trial'/exp OR 'phase 2 clinical trial'/exp OR 'phase 3 clinical trial'/exp OR 'phase 4 clinical trial'/exp OR 'double-blind procedure'/exp OR 'single-blind procedure'/exp OR 'multicenter study'/exp OR 'crossover procedure'/exp OR 'comparative study'/exp OR 'placebo'/exp OR 'randomization'/exp OR 'random*':ab,ti OR 'randomized controlled trial*' OR 'rct':ab,ti OR 'randomized trial*':ab,ti OR 'controlled trial*':ab,ti OR 'controlled stud*':ab,ti OR 'clinical trial*':ab,ti OR 'clinical stud*':ab,ti OR 'controlled clinical stud*':ab,ti OR 'controlled clinical trial*':ab,ti OR 'single-blind*':ab,ti OR 'double-blind*':ab,ti OR 'treble-blind*':ab,ti OR 'triple-blind*':ab,ti OR 'cross-over*':ab,ti OR 'single blind*':ab,ti OR 'double blind*':ab,ti OR 'treble blind*':ab,ti OR 'triple blind*':ab,ti OR 'cross over*':ab,ti OR 'placebo*':ab,ti OR 'factorial*':ab,ti OR 'assign*':ab,ti OR 'allocat*':ab,ti OR 'control*':ab,ti OR 'trial*':ab,ti OR 'volunteer*':ab,ti)

Database: Cochrane Central Register of Controlled Trials

Result: 860

Search strings: #1 MeSH descriptor: [COVID-19] explode all trees

#2 MeSH descriptor: [SARS-CoV-2] explode all trees

#3 #1 or #2

#4 ("COVID-19" or "COVID 19" or "SARS-CoV-2" or "SARS Coronavirus 2" or "Severe Acute Respiratory Syndrome Coronavirus 2" or "2019-nCoV" or "2019 nCoV" or "nCoV-19" or "nCoV 19" or "Coronavirus Disease-19" or "Coronavirus Disease 19" or "Coronavirus Disease 2019" or "Coronavirus Disease-2019 Virus" or nCoV or "Novel Coronavirus"):ti,ab,kw (Word variations have been searched)

**#5 #3 or #4**

#6 MeSH descriptor: [COVID-19 Vaccines] explode all trees

#7 MeSH descriptor: [Vaccines] in all MeSH products

#8 #6 or #7

#9 (vaccin* or immunis* or immuniz*):ti,ab,kw (Word variations have been searched)

**#10 #8 or #9**

**#11** (safety or "adverse event" or "adverse reactions" or "side effects" or tolerance or tolerabilit* or reactogenicity or effectiveness or efficacy or immunogenicity or outcome*):ti,ab,kw (Word variations have been searched)

#12 MeSH descriptor: [Aged] explode all trees

#13 MeSH descriptor: [Aged, 80 and over] explode all trees

#14 MeSH descriptor: [Frail Elderly] explode all trees

#15 #12 or #13 or #14

#16 (older* or "older adult" or olds or elder* or aging or ageing or aged or ag*ing or seni* or geriatr* or gerontolog* or "late-life" or "late life" or vulnerab*):ti,ab,kw (Word variations have been searched)

**#17 #15 or #16**

**#18 #5 or #10 or #11 or #17**

Database: Web of Science

Result: 741

Search strings: (TS=("COVID-19" OR "COVID 19" OR "SARS-CoV-2" OR "SARS Coronavirus 2" OR "Severe Acute Respiratory Syndrome Coronavirus 2" OR "2019-nCoV" OR "2019 nCoV" OR "nCoV-19" OR "nCoV 19" OR "Coronavirus Disease-19" OR "Coronavirus Disease 19" OR "Coronavirus Disease 2019" OR "Coronavirus Disease-2019 Virus" OR "nCoV" OR "Novel Coronavirus")) **AND** (TS=("vaccin*" OR "immunis*" OR "immuniz*")) **AND** (ALL=("safety" OR "adverse event*" OR "adverse reaction*" OR "side effects" OR "tolerance" OR "tolerabilit*" OR "reactogenicity" OR "effectiveness" OR "efficacy" OR "immunogenicity" OR "outcome*")) **AND** (ALL=("Aged" OR "Aged, 80 and over" OR "Frail Elderly" OR "older*" OR "older adult*" OR "olds" OR "elder*" OR "aging" OR "ageing" OR "senior*" OR "geriatr*" OR "gerontolog*" OR "late-life" OR "late life" OR "vulnerab*")) **AND** (TS=("Controlled clinical trial*" OR "Multicenter Stud*" OR "Comparative Stud*" OR "Cross-Over Studies" OR "Double-Blind Method" OR "Single-Blind Method" OR "Random Allocation" OR "Placebo*" OR "random*" OR "randomized controlled trial*" OR "RCT" OR "randomized trial*" OR "controlled clinical stud*" OR **"**controlled clinical trial*" OR "controlled trial*" OR "controlled stud*" OR "clinical trial*" OR "clinical stud*" OR "single-blind*" OR "double-blind*" OR "treble-blind*" OR "triple-blind*" OR "cross-over*" OR "single blind*" OR "double blind*" OR "treble blind*" OR "triple blind*" OR "cross over*" OR "factorial*" OR "assign*" OR "allocat*" OR "control*" OR "trial*" OR "volunteer*"))

Indexes = SCI-EXPANDED, SSCI, A&HCI, CPCI-S, CPCI-SSH, BKCI-S, BKCI-SSH, ESCI, CCR-EXPANDED, IC Timespan = All years

**A**

**B**

**C**

**D**

**E**

**F**

**G**

**H**

**Supplementary Figure S1.** The forest plot of meta-analysis. **(A)** Vaccine efficacy for various vaccine types; **(B)** Vaccine efficacy for symptomatic COVID-19; **(C)** Vaccine efficacy for severe COVID-19. **(D)** Neutralizing antibody seroconversion rates transformed by double arcsine . **(E)** Total adverse events. **(F)** Systemic adverse events. **(G)** Local adverse events. **(H)** Adverse events after the first dose versus the second dose of vaccination. †For the BNT162b1 vaccine; ‡for the BNT162b2 vaccine. *COVID-19 vaccines on first vaccination; **COVID-19 vaccines on second vaccination; ***COVID-19 vaccines on third vaccination.

**A**

**B**

**C**

**D**

**E**

**F**

**Supplementary Figure S2.** The forest plots for the specific populations (aged ≥ 60, ≥ 65 or ≥ 75 years). **(A)** Vaccine efficacy. **(B)** Neutralizing antibody seroconversion rates. **(C)** The GMT values of log-transformed neutralizing antibody. **(D)** Total adverse events. **(E)** Systemic adverse events. **(F)** Local adverse events. †For the BNT162b1 vaccine; ‡for the BNT162b2 vaccine. *COVID-19 vaccines on first vaccination; **COVID-19 vaccines on second vaccination; ***COVID-19 vaccines on third vaccination.

**A**

**B**

**C**

**D**

**E**

**Supplementary Figure S3.** The funnel plots after trim-and-fill analysis. **(A)** Vaccine efficacy. After adding these two studies to the meta-analysis, the pooled vaccine efficacy became 74.18% (95% CI: 53.70−85.60). **(B)** Neutralizing antibody seroconversion rates. With the addition of these four studies, the estimated combined seroconversion rate changed to 89.08% (95% CI: 82.07−94.50). **(C)** Total adverse events. The summary risk ratios estimates of total AEs changed to 1.82 (95% CI: 1.58−2.09) after adding five hypothetical studies. **(D)** Systemic adverse events. **(E)** Local adverse events.

**A**

**B**

**C**

**D**

**E**

**Supplementary Figure S4.** Sensitivity analysis results. **(A)** Vaccine efficacy. **(B)** Neutralizing antibody seroconversion rates. **(C)** Total adverse events. **(D)** Systemic adverse events. **(E)** Local adverse events. †For the BNT162b1 vaccine; ‡for the BNT162b2 vaccine. *COVID-19 vaccines on first vaccination; **COVID-19 vaccines on second vaccination; ***COVID-19 vaccines on third vaccination.

**A**

**B**

**C**

**D**

**E**

**Supplementary Figure S5.** The Galbraith plot of meta-analysis. **(A)** Vaccine efficacy. **(B)** Neutralizing antibody seroconversion rates. **(C)** Total adverse events. **(D)** Systemic adverse events. **(E)** Local adverse events.

**A**

**B**

**Supplementary Figure S6.** The results of literature quality (risk of bias) assessment. **(A)** Risk of bias summary. **(B)** Risk of bias graph.

**Supplementary Table S1.** Characteristics of included studies on the safety of COVID-19 vaccines.

| **Study** | **Vaccines** | **Administration (dosage, no. of doses)** | **Age range** | **No. of participants (N 1/N 2)** | **Country** | **Study types**  **(phase, no. of centers, blind)** | **RR of total AEs (95% CI)** |
| --- | --- | --- | --- | --- | --- | --- | --- |
| Asano 2022 | ChAdOx1-S-(AZD1222) | 5 × 10^10^ VP, 2 | ≥ 56 | 96/32 | Japan | Ⅰ/Ⅱ, 5, double-blind | − |
| Bueno 2021 | CoronaVac | 3 µg, 2 | ≥ 60 | 25/12 | Chile | Ⅲ, 8, observer-blinded | − |
| Chu 2021 | mRNA-1273 | 50 µg/100 µg, 2 | ≥ 55 | 200/99 | the United States | Ⅱ, 8, observer-blinded | 1.79 (1.39, 2.29)*  2.23 (1.72, 2.88)** |
| Falsey 2021 | ChAdOx1-S-(AZD1222) | 5 × 10^10^ VP, 2 | ≥ 65 | 698/350 | the United States, Chile, Peru | Ⅲ, 88, double-blind | − |
| Formica 2021 | NVX-CoV2373 | 5 µg/25 µg, 2 | ≥ 60 | 463/114 | the United States, Australia | Ⅱ, 17, observer-blinded | − |
| Guo 2021 | WIBP COVID-19 vaccine | 2.5 µg/5 µg/10 µg, 3 | ≥ 60 | 252/84 | China | Ⅰ/Ⅱ, 2, double-blind | 0.55 (0.30, 1.01) |
| Haranaka 2021 | BNT162b2 | 30 µg, 2 | ≥ 65 | 22/8 | Japan | Ⅰ/Ⅱ, 2, observer-blind | − |
| Hsieh 2021 | MVC-COV1901 | 15 µg, 2 | ≥ 65 | 720/118 | China | Ⅱ, 11, double-blind | − |
| Khobragade 2022 | ZyCoV-D | 2 mg, 3 | ≥ 60 | 924/923 | India | Ⅲ, 49, double-blind | 0.39 (0.16, 0.93)*  0.76 (0.34, 1.73)**  1.39 (0.62, 3.11)*** |
| Li 2021 | BNT162b1 | 10 µg/30 µg, 2 | ≥ 65 | 48/24 | China | Ⅰ, 1,  double-blind | 11.00 (2.91, 41.58) |
| Liu 2021 | KCONVAC | 5 µg/10 µg, 3 | ≥ 60 | 200/50 | China | Ⅱ, 3, double-blind | 1.75 (0.41, 7.45) |
| Logunov 2021 | Gam-COVID-Vac | 1 × 10^11^ VP, 2 | > 60 | 1029/340 | Russia | Ⅲ, 25, double-blind | − |
| Masuda 2022 | mRNA-1273 | 100 mg, 2 | ≥ 65 | 50/10 | Japan | Ⅰ/Ⅱ, 2, observer-blinded | 1.76 (0.94, 3.30)*  6.53 (1.01, 42.40)** |
| Meng 2021 | Recombinant COVID-19 vaccine (Sf9 cells) | 20 µg/40 µg, 3 | ≥ 60 | 453/98 | China | Ⅰ/Ⅱ, 1, double-blind | 1.11 (0.51, 2.43)*  0.87 (0.39, 1.92)** |
| Ramasamy 2021 | ChAdOx1-S-(AZD1222) | 3.5−6.5 × 10^10^ VP, 2 | ≥ 55 | 155/40 | the UK | Ⅱ, 20, observer-blinded | 1.59 (1.13, 2.23)*  1.61 (1.01, 2.38)** |
| Richmond 2021 | SCB-2019 | 3 µg/9 µg/30 µg, 2 | ≥ 55 | 48/12 | Australia | Ⅰ, 1,  double-blind | − |
| Sadoff 2021 | Ad26.COV2.S | 5 × 10^10^/  1 × 10^11^ VP, 1 | ≥ 65 | 322/81 | Belgium, the United States | Ⅰ/Ⅱ a, 12, double-blind | 2.26 (1.59, 3.23) |
| Sahly 2021 | mRNA-1273 | 100 µg, 2 | ≥ 65 | 3760/3749 | the United States | Ⅲ, 99, observer-blinded | 1.97 (1.89, 2.05)*  2.45 (2.35, 2.56)** |
| Shu 2021 | Recombinant SARS-CoV-2 Fusion Protein Vaccine (V-01) | 10 µg/25 µg/50 µg, 2 | ≥ 60 | 360/80 | China | Ⅱ, 1, double-blind | 0.73 (0.38, 1.43)*  2.27 (0.13, 39.50)** |
| Walsh 2020 | BNT162b1, BNT162b2 | 10 µg/20 µg/30 µg, 2 | ≥ 65 | 36/9†, 36/9‡ | the United States | Ⅰ, ≤ 4, observer-blinded | 1.06 (0.47, 2.38)†  0.75 (0.18, 3.11)‡ |
| Wu 2021§ | CoronaVac | 1.5 µg/3 µg/6 µg, 2 | ≥ 60 | 347/74 | China | Ⅰ/Ⅱ, 1, double-blind | 0.89 (0.49, 1.64)*  1.59 (0.65, 3.89)** |
| Xia 2021 | BBIBP-CorV | 2 µg/4 µg/8 µg, 2 | ≥ 60 | 72/24 | China | Ⅰ, 1,  double-blind | 2.00 (0.48, 8.31) |
| Zeng 2022§ | CoronaVac | 1.5 µg/3 µg/6 µg, 3 | ≥ 60 | 256/47 | China | Ⅱ, 1, double-blind | 0.86 (0.26, 2.87) |
| Zhang 2021 | Recombinant SARS-CoV-2 Fusion Protein Vaccine (V-01) | 10 µg/25 µg/50 µg, 2 | ≥ 60 | 72/24 | China | Ⅰ, 1,  double-blind | 2.34 (0.13, 41.64)*  0.78 (0.03, 18.41)** |
| Zhu 2021 | Ad5-nCoV | 5 × 10^10^/  1 × 10^11^ VP, 2 | ≥ 56 | 200/50 | China | Ⅱ b, 1, double-blind | 3.92 (1.27, 12.07)*  1.71 (0.77, 3.80)** |

Vp = viral particles; AEs = adverse events; N1 = the sample size of vaccination group; N2 = the sample size of control group. §Two articles are from the same trial. †For the BNT162b1 vaccine; ‡for the BNT162b2 vaccine. *Indicated the RR estimates of total AEs after the first injection; **Indicated the RR estimates of total AEs after the second injection; ***Indicated the RR estimates of total AEs after the third injection.

**Supplementary Table S2.** Combined seroconversion rates of spike-specific and RBD-specific IgG to different vaccine types.

|  | **No. of Studies** | **No. of participants (n/N)** | **Estimated seroconversion rate (95% CI)** | **Heterogeneity I^2^ (%)** | **Test of heterogeneity (*p* value)** |
| --- | --- | --- | --- | --- | --- |
| **Seroconversion of spike-specific IgG to different vaccine types** | | | | | 0.654 |
| Adenovirus vector vaccines | 2 | 139/245 | 98.25% (92.19, 99.97) | 81.71 |  |
| Subunit vaccines | 3 | 456/462 | 98.44% (96.42, 99.64) | 39.79 |  |
| mRNA vaccines | 2 | 95/95 | 99.49% (97.09, 99.92) | 0.00 |  |
| **Overall** | **7** | **790/802** | **98.54% (97.13, 99.49)** | **42.73** |  |
| **Seroconversion of RBD-specific IgG to different vaccine types** | | | | | ˂ 0.001 |
| Adenovirus vector vaccines | 3 | 276/304 | 91.25% (86.50, 95.07) | 34.36 |  |
| Inactivated virus vaccines | 1 | 178/179 | 99.19% (97.36, 99.97) | − |  |
| Subunit vaccines | 2 | 321/348 | 91.42% (64.02, 99.77) | 97.42 |  |
| mRNA vaccines | 1 | 46/46 | 99.46% (95.39, 99.51) | − |  |
| **Overall** | **7** | **821/877** | **94.72% (88.35, 98.68)** | **91.27** |  |

RBD: Receptor-binding domain; n = the number of participants with seroconversion; N = total number of vaccinated participants assessing spike-spike specific antibodies or RBD specific antibodies.

**Supplementary Table S3.** Subgroup analysis of the total adverse events of COVID-19 vaccines across variables.

| **Subgroups** | **No. of Studies** | **Reactions/total** | | **RR (95% CI)** | **Heterogeneity I^2^ (%)** | ***P* value** |
| --- | --- | --- | --- | --- | --- | --- |
|  |  | **Vaccination** | **Control** |  |  |  |
| **Continent** |  |  |  |  |  | 0.021 |
| Asia | 12 | 494/6341 | 119/3511 | 1.25 (0.91, 1.71) | 51.75 |  |
| Europe | 1 | 218/308 | 35/79 | 1.60 (1.24,2.06) | 0.00 |  |
| North America | 3 | 6701/7923 | 2867/7614 | 2.04 (1.73, 2.40) | 91.38 |  |
| Multi-continent | 1 | 207/322 | 23/81 | 2.26 (1.59, 3.23) | − |  |
| **Study design** |  |  |  |  |  | 0.017 |
| Double-blind | 12 | 625/6564 | 136/3572 | 1.25 (0.89, 1.75) | 61.75 |  |
| Observer-blinded | 5 | 6995/8330 | 3008/7713 | 1.95 (1.69, 2.25) | 86.19 |  |
| **Vaccine type** |  |  |  |  |  | ˂ 0.001 |
| Adenovirus vector vaccines | 3 | 513/1030 | 67/260 | 1.84 (1.50, 2.27) | 0.00 |  |
| DNA vaccines | 1 | 31/2678 | 41/2664 | 0.75 (0.37, 1.54) | 55.06 |  |
| Inactivated virus vaccines | 5 | 147/1472 | 37/351 | 0.94 (0.63, 1.41) | 17.33 |  |
| Subunit vaccines | 3 | 108/1644 | 24/352 | 0.91 (0.60, 1.37) | 0.00 |  |
| mRNA vaccines | 5 | 6821/8070 | 2975/7658 | 2.08 (1.77, 2.45) | 87.79 |  |
| **No. of injections** |  |  |  |  |  | 0.454 |
| First dose | 13 | 3811/6963 | 1693/5264 | 1.52 (1.20, 1.92) | 70.83 |  |
| Second dose | 10 | 3732/6282 | 1420/4953 | 1.80 (1.39, 2.34) | 58.96 |  |
| Third dose | 2 | 28/1125 | 13/910 | 1.20 (0.61, 2.34) | 0.00 |  |
| Mixed doses | 3 | 49/524 | 18/158 | 1.02 (0.40, 2.59) | 51.59 |  |

**Supplementary Table S4.** The results of univariate and multivariate meta-regression analyses on the neutralizing antibody seroconversion rate.

| **Variables** | | **Coefficient** | | **95% CI** | | **Std. Err** | | ***P* value** | |
| --- | --- | --- | --- | --- | --- | --- | --- | --- | --- |
| **Univariate Analysis** | | | | | | | | | |
| **Continent** | Asia | | ref | | ref | | ref | | ref |
|  | Europe | | 0.2501 | | (−0.8522, 1.3524) | | 0.5624 | | 0.657 |
|  | North America | | 0.5271 | | (−0.3458, 1.4001) | | 0.4454 | | 0.237 |
|  | Oceania | | 0.1233 | | (−0.8193, 1.0660) | | 0.4809 | | 0.798 |
|  | Multi-continent | | 0.1476 | | (−0.5024, 0.7976) | | 0.3316 | | 0.656 |
| **Study design** | Double-blind | | ref | | ref | | ref | | ref |
|  | Observer-blinded | | 0.4382 | | (−0.0761, 0.9526) | | 0.2624 | | 0.095 |
| **Vaccine type** | Adenovirus vector vaccines | | ref | | ref | | ref | | ref |
|  | Inactivated virus vaccines | | 0.6947 | | (0.2012, 1.1882) | | 0.2518 | | 0.006 |
|  | Subunit vaccines | | 0.5559 | | (0.0575, 1.0544) | | 0.2543 | | 0.029 |
|  | mRNA vaccines | | 0.7848 | | (0.2099, 1.3597) | | 0.2933 | | 0.007 |
| **No. of doses** | 1 dose | | ref | | ref | | ref | | ref |
|  | 2 doses | | 0.8818 | | (0.2858, 1.4778) | | 0.3041 | | 0.004 |
|  | 3 doses | | 0.6912 | | (0.0217, 1.3607) | | 0.3416 | | 0.043 |
| **Days of immunoassay after the last vaccination** | 14 days | | ref | | ref | | ref | | ref |
|  | 21 days | | −0.2520 | | (−1.1462, 0.6422) | | 0.4562 | | 0.581 |
|  | 28 days | | −0.3774 | | (−1.0140, 0.2592) | | 0.3248 | | 0.245 |
| **Multivariate Analysis** | | | | | | | | | |
| **Study design** | Double-blind | | ref | | ref | | ref | | ref |
|  | Observer-blinded | | −1.5253 | | (−3.4130, 0.3623) | | 0.9631 | | 0.113 |
| **Vaccine type** | Adenovirus vector vaccines | | ref | | ref | | ref | | ref |
|  | Inactivated virus vaccines | | 0.3531 | | (−0.3702, 1.0764) | | 0.3690 | | 0.339 |
|  | Subunit vaccines | | 0.2234 | | (−0.4721, 0.9189) | | 0.3549 | | 0.529 |
|  | mRNA vaccines | | 2.0968 | | (−0.0725, 4.2661) | | 1.1068 | | 0.058 |
| **No. of doses** | 1 dose | | ref | | ref | | ref | | ref |
|  | 2 doses | | 1.3157 | | (0.2870, 2.3444) | | 0.5248 | | 0.012 |
|  | 3 doses | | 1.2012 | | (0.1164, 2.2859) | | 0.5535 | | 0.030 |
| **Days of immunoassay after the last vaccination** | 14 days | | ref | | ref | | ref | | ref |
|  | 21 days | | −2.1762 | | (−5.0798, 0.7274) | | 1.4815 | | 0.142 |
|  | 28 days | | −0.2413 | | (−1.3162, 0.8335) | | 0.5484 | | 0.660 |

**Supplementary Table S5.** The results of univariate and multivariate meta-regression analyses on total adverse events of COVID-19 vaccines.

| **Variables** | | **Coefficient** | **95% CI** | **Std. Err** | ***p*-Value** |
| --- | --- | --- | --- | --- | --- |
| **Univariate Analysis** | | | | | |
| **Continent** | Asia | ref | ref | ref | ref |
|  | Europe | 0.3346 | (−0.0822, 0.7513) | 0.2126 | 0.116 |
|  | North America | 0.5676 | (0.2796, 0.8557) | 0.1470 | ˂ 0.001 |
|  | Multi-continent | 0.6828 | (0.1432, 1.2223) | 0.2753 | 0.013 |
| **Study design** | Observer-blinded | ref | ref | ref | ref |
|  | Double-blind | −0.4518 | (−0.7237, −0.1800) | 0.1387 | 0.001 |
| **Vaccine type** | mRNA vaccines | ref | ref | ref | ref |
|  | Adenovirus vector vaccines | −0.1153 | (−0.4142, 0.1836) | 0.1525 | 0.450 |
|  | DNA vaccines | −1.0021 | (−1.5364, −0.4678) | 0.2726 | ˂ 0.001 |
|  | Inactivated virus vaccines | −0.8102 | (−1.2209, −0.3995) | 0.2096 | ˂ 0.001 |
|  | Subunit vaccines | −0.8245 | (−1.2986, −0.3504) | 0.2419 | 0.001 |
| **No. of injections** | Second dose | ref | ref | ref | ref |
|  | First dose | −0.1490 | (−0.5129, 0.2148) | 0.1856 | 0.422 |
|  | Third dose | −0.4103 | (−1.2501, 0.4295) | 0.4285 | 0.338 |
|  | Mixed doses | −0.7177 | (−1.4383, 0.0029) | 0.3677 | 0.051 |
| **Multivariate Analysis** | | | | | |
| **Continent** | Asia | ref | ref | ref | ref |
|  | Europe | 1.4250 | (−0.1993, 3.0493) | 0.8287 | 0.086 |
|  | North America | −0.0190 | (−0.6258, 0.5878) | 0.3096 | 0.951 |
|  | Multi-continent | 0.1475 | (−0.6077, 0.9027) | 0.3853 | 0.702 |
| **Study design** | Observer-blinded | ref | ref | ref | ref |
|  | Double-blind | 1.7200 | (0.2578, 3.1821) | 0.7460 | 0.021 |
| **Vaccine type** | mRNA vaccines | ref | ref | ref | ref |
|  | Adenovirus vector vaccines | −1.7283 | (−3.2156, −0.2410) | 0.7588 | 0.023 |
|  | DNA vaccines | −2.9465 | (−4.3930, −1.5000) | 0.7380 | ˂ 0.001 |
|  | Inactivated virus vaccines | −2.5108 | (−3.9292, −1.0924) | 0.7237 | 0.001 |
|  | Subunit vaccines | −2.5632 | (−3.9604, −1.1660) | 0.7129 | ˂ 0.001 |
| **No. of injections** | Second dose | ref | ref | ref | ref |
|  | First dose | −0.2186 | (−0.3440, −0.0932) | 0.0640 | 0.001 |
|  | Third dose | 0.3746 | (−0.4282, 1.1775) | 0.4096 | 0.360 |
|  | Mixed doses | −0.3722 | (−1.0957, 0.3513) | 0.3691 | 0.313 |

**Supplementary Table S6.** Evaluation of the quality of evidence using GRADE system for primary outcomes.*

| **Primary outcomes** | **Study limitations** | **Imprecision** | **Heterogeneity and inconsistency** | **Indirectness** | **Publication bias** | **Certainty of the evidence**  **(GRADE)** |
| --- | --- | --- | --- | --- | --- | --- |
| Vaccine efficacy | **44.4% (4/9) of the estimate from studies at high risk, and 55.6% (5/9) at unclear.** | 79.49% (95% CI: 60.55−89.34) | High heterogeneity according to I^2^ (87.37%), but capable of being reasonably interpreted. No serious inconsistency. | No serious indirectness. | Undetectable by the routine method or less likely to be present. | **Moderate** (Downgrade by one level due to study limitations. |
| nAb GMT | **33.3% (6/18) of the estimate from studies at high risk, 61.1% (11/18) at unclear, and 5.6% (1/18) at low risk.** | SMD 3.56 (95% CI: 2.80−4.31) | **High heterogeneity according to I^2^ (97.37%). No serious inconsistency.** | No serious indirectness. | **Publication bias existed and was strongly suspected.** | **Very low** (Downgrade by three levels due to study limitations, heterogeneity, and publication bias). |
| nAb seroconversion rate | **47.4% (9/19) of the estimate from studies at high risk, 47.4% (9/19) at unclear, and 5.2% (1/19) at low risk.** | 92.64% (95% CI: 86.77−96.91) | **High heterogeneity according to I^2^ (95.03%). No serious inconsistency.** | No serious indirectness. | Undetectable by the routine method or less likely to be present. | **Low** (Downgrade by two levels due to study limitations and heterogeneity). |
| Total adverse events | **52.9% (9/17) of the estimate from studies at high risk, 41.2% (7/17) at unclear, and 5.9% (1/17) at low risk.** | RR 1.59 (95% CI: 1.38−1.83) | **High heterogeneity according to I^2^ (80.33%). No serious inconsistency.** | No serious indirectness. | **Publication bias existed and was strongly suspected.** | **Very low** (Downgrade by three levels due to study limitations, heterogeneity, and publication bias). |
| Systemic adverse events (any) | **50.0% (8/16) of the estimate from studies at high risk, 43.8% (7/16) at unclear, and 6.2% (1/16) at low risk.** | RR 1.55 (95% CI: 1.30−1.85) | **High heterogeneity according to I^2^ (89.86%). No serious inconsistency.** | No serious indirectness. | Undetectable by the routine method and the funnel plot showed symmetry. | **Low** (Downgrade by two levels due to study limitations and heterogeneity). |
| Local adverse events (any) | **47.1% (8/17) of the estimate from studies at high risk, 47.1% (8/17) at unclear, and 5.8% (1/17) at low risk.** | RR 3.42 (95% CI: 2.74−4.28) | **High heterogeneity according to I^2^ (85.44%). No serious inconsistency.** | No serious indirectness. | Undetectable by the routine method and the funnel plot showed symmetry. | **Low** (Downgrade by two levels due to study limitations and heterogeneity). |

*Guyatt GH, Oxman AD, Vist GE, Kunz R, Falck-Ytter Y, Alonso-Coello P, et al. GRADE Working Group. GRADE: an emerging consensus on rating quality of evidence and strength of recommendations. BMJ (2008) 336(7650):924-926. Epub 2008/04/26. doi: 10.1136/bmj.39489.470347.AD.

**High certainty:** We are very confident that the true effect lies close to that of the estimate of the effect.

**Moderate certainty:** We are moderately confident in the effect estimate: The true effect is likely to be close to the estimate of the effect, but there is a possibility that it is substantially different.

**Low certainty:** Our confidence in the effect estimate is limited: The true effect may be substantially different from the estimate of the effect.

**Very low certainty:** We have very little confidence in the effect estimate: The true effect is likely to be substantially different from the estimate of effect.

nAb = neutralizing antibody.
